# Integrative computational approaches identify haptoglobin inhibitors to modulate erythrocyte sedimentation rate in trauma-linked inflammatory and haematological malignancies

**DOI:** 10.3389/fchem.2025.1611972

**Published:** 2025-06-18

**Authors:** Abdulaziz H. Al Khzem, Shaban Ahmad

**Affiliations:** ^1^ Department of Pharmaceutical Chemistry, College of Pharmacy, Imam Abdulrahman Bin Faisal University, Dammam, Saudi Arabia; ^2^ Department of Computer Science, Jamia Millia Islamia, New Delhi, India

**Keywords:** haptoglobin, drug design and discovery, computational medicinal chemistry, molecular docking, binding free energy

## Abstract

Elevated levels of haptoglobin are commonly observed in conditions characterised by an increased erythrocyte sedimentation rate which are acute-phase reactants. These conditions include infection, trauma, inflammation, hepatitis, amyloidosis, collagen diseases, lymphoma, leukaemia, as well as obstructive and biliary diseases. However, no significant drugs are currently available to manage these conditions, making therapeutic intervention crucial effectively. In this study, we performed an extensive screening of the DrugBank database against the human haptoglobin protein (PDB ID: 4X0L) using High-Throughput Virtual Screening (HTVS), Standard Precision and Extra Precision (XP) docking methods, followed by pose processing with Molecular Mechanics Generalised Born Surface Area (MM/GBSA) calculations. This led to the identification of five potential inhibitors: L-histidinol phosphate (DB03997), L-gluconic acid (DB04304), 4-bromo-3-(carboxymethoxy)-5-(4-hydroxyphenyl)thiophene-2-carboxylic acid (DB07197), 3-O-methylfructose (DB02438), and glutamine hydroxamate (DB02446), with docking scores ranging from −7.96 to −5.58 kcal/mol and MM/GBSA scores between −26.23 and −1.00 kcal/mol. The study also included Density Functional Theory computations and pharmacokinetic profiling to assess these compounds’ suitability further, revealing promising results. Additionally, we conducted molecular interaction fingerprint analysis, revealing key residues involved in interactions, including 10LYS (Basic), 8LEU (non-polar), 7ASP (Acidic), and 7THR (Polar), indicating a mixed interaction profile. A 5 ns WaterMap analysis was used to identify optimal hydration sites and interaction patterns. Moreover, a 100 ns molecular dynamics (MD) simulation using the TIP3P water model in the NPT ensemble confirmed the stability of the protein-ligand complexes, with acceptable deviations, fluctuations, and intermolecular interactions. MM/GBSA calculations on the simulation trajectories supported these findings by providing binding free energy and complex energy estimations for all protein-ligand complexes. Although these findings provide compelling computational evidence for haptoglobin inhibition, experimental studies must confirm its effectiveness before human use.

## 1 Introduction

Haptoglobin (Hp) is a crucial glycoprotein synthesised predominantly in the liver and secreted into the plasma, where it plays an essential role in haemoglobin (Hb) scavenging, immune modulation, and various inflammatory pathways (Schaer et al., 2014; Delanghe et al., 2024). It is a key component of the acute-phase response, acting as a protective mechanism against oxidative damage caused by free haemoglobin released from erythrocytes during haemolysis. Hp is encoded by the HP gene, which exhibits polymorphism, primarily classified into three primary phenotypic forms: Hp1-1, Hp2-1, and Hp2-2. These phenotypic variations influence its biological functions and its interaction with other biomolecules (Montecinos et al., 2019; Roh et al., 2025). Haptoglobin forms a stable complex with free haemoglobin, rapidly cleared from circulation via CD163-mediated endocytosis in monocytes and macrophages (Schaer et al., 2006). This mechanism is crucial in preventing oxidative stress and tissue damage that would otherwise result from the peroxidative activity of free heme. Hp also modulates inflammation by regulating immune responses and influencing cytokine release, contributing to its relevance in several physiological and pathological conditions (Skytthe et al., 2020; Delanghe et al., 2024). The pathways involving haptoglobin are intricately linked to its haemoglobin-binding function and its broader implications in immune responses. The Hp-Hb complex formation triggers a cascade of cellular and molecular events leading to endocytosis and lysosomal degradation of the complex in macrophages (Nielsen and Moestrup, 2009). This process prevents the generation of free heme and iron-mediated oxidative stress. The binding of haptoglobin to haemoglobin induces conformational changes that enhance its recognition by CD163, a scavenger receptor expressed in monocytes and macrophages (de Oliveira et al., 2022). Once internalised, the complex is degraded in lysosomes, and the iron component is stored within ferritin or exported via ferroprotein. This pathway plays a significant role in iron homeostasis, preventing iron overload and associated toxicity (Galy et al., 2024). Additionally, haptoglobin has been implicated in modulating inflammatory pathways through its interaction with various cytokines and immune cells. It can suppress prostaglandin synthesis, inhibit neutrophil migration, and modulate the activity of Toll-like receptors (TLRs), thereby regulating innate immunity. Furthermore, its involvement in lipid metabolism and high-density lipoprotein (HDL) function suggests a broader role in cardiovascular health and disease pathogenesis (Knutson, 2017; Singh et al., 2025).

In blood circulation, haptoglobin is a critical scavenger protein, ensuring free haemoglobin does not exert toxic effects on vascular integrity. During intravascular haemolysis, red blood cells rupture and release haemoglobin into circulation, where it can undergo oxidation and generate reactive oxygen species (ROS) (Van Avondt et al., 2019; Orrico et al., 2023). The binding of Hp to Hb neutralises these oxidative effects and facilitates the complex’s rapid clearance, thus protecting endothelial cells from oxidative damage. Haptoglobin also interacts with coagulation pathways, influencing clot formation and fibrinolysis. It has been shown to affect platelet function and the activity of coagulation factors, thereby modulating thrombotic and haemorrhagic tendencies. Additionally, haptoglobin plays a vital role in trauma and accidents, where massive haemolysis can lead to systemic inflammation, disseminated intravascular coagulation (DIC), and multi-organ failure (Gbotosho et al., 2021). By sequestering free haemoglobin, haptoglobin mitigates these complications and contributes to haemostatic balance. Trauma-related inflammation refers to the systemic inflammatory response triggered by physical injury, such as trauma from accidents, burns, or surgeries (Brøchner and Toft, 2009). This inflammation can lead to multiple physiological changes, including the elevation of several acute-phase proteins, among which haptoglobin is a key player. The body’s response to trauma involves a complex cascade of immune and metabolic reactions to repair tissue damage and prevent infection. One of the most commonly measured indicators of systemic inflammation is the erythrocyte sedimentation rate (ESR), a clinical test that measures the rate at which red blood cells settle at the bottom of a test tube (Tishkowski and Gupta, 2020). Elevated ESR values indicate increased systemic inflammation, often observed in response to trauma and other inflammatory conditions. Haptoglobin is upregulated during trauma-induced inflammation as part of the acute-phase response, which is triggered by the release of inflammatory cytokines such as interleukins and tumour necrosis factor-alpha (TNF-α) (Tishkowski and Gupta, 2020). In this context, the relationship between haptoglobin and ESR is significant: as an acute-phase protein, haptoglobin levels rise in response to systemic inflammation, which often parallels the increase in ESR levels. Both markers indicate the extent of tissue injury and the body’s inflammatory response. The binding of haptoglobin to free haemoglobin, released during red blood cell lysis, further reduces oxidative damage and modulates the inflammatory process. Elevated haptoglobin levels are thought to correlate with ESR in trauma-related inflammation, serving not only as an indicator of inflammation but also play a protective role by preventing oxidative stress and tissue damage. These interactions are central to the immune response following trauma, and a better understanding of this relationship can provide insight into the potential therapeutic applications of haptoglobin modulators in managing inflammation following traumatic events (Baxter-Parker, 2019). During inflammatory states, its levels increase as part of the acute-phase response, helping to modulate excessive immune activation and tissue damage. Haptoglobin’s role extends to pathological conditions such as lymphoma and leukaemia, where its expression and function are altered due to disease progression (Wang et al., 2021). Aberrant haptoglobin levels can be a disease status and prognosis biomarker in haematological malignancies. Increased serum haptoglobin levels have been observed in various cancers, including lymphoma and leukaemia, reflecting its role in the tumour microenvironment (Delanghe et al., 2024). Haptoglobin has increasingly been recognised for its involvement in cancer biology. Elevated levels of haptoglobin are commonly observed in various malignancies, including lymphoma, leukaemia, and solid tumours such as breast, prostate, and colorectal cancers (Naryzny and Legina, 2021). The role of haptoglobin in malignancy is multifaceted, influencing both tumour progression and the microenvironment. One of the primary functions of haptoglobin in cancer is its interaction with the immune system. Haptoglobin modulates inflammatory responses, which are crucial in the pathogenesis of cancer. In particular, haptoglobin’s anti-inflammatory effects can suppress immune surveillance, aiding in tumour immune evasion. It has been shown that haptoglobin can alter cytokine profiles in the tumour microenvironment, promoting tumour growth and metastasis (Lu et al., 2016). For example, in lymphoma and leukaemia, high levels of haptoglobin correlate with poor prognosis, potentially due to its role in suppressing anti-tumour immune responses and fostering an immunosuppressive environment. Additionally, haptoglobin has been implicated in angiogenesis, the process by which new blood vessels form to supply the growing tumour with nutrients and oxygen (Lu et al., 2016). By modulating the balance of reactive oxygen species (ROS) in the tumour microenvironment, haptoglobin can influence endothelial cell function and blood vessel formation, facilitating tumour growth and metastasis (Costa et al., 2014). Moreover, haptoglobin’s role in iron metabolism, particularly its ability to scavenge free haemoglobin, may also contribute to iron homeostasis in tumour cells, providing the conditions for uncontrolled cell proliferation. Given these multifaceted roles, haptoglobin is increasingly considered a potential malignancy biomarker and therapeutic target (Costa et al., 2014). Modulating haptoglobin activity by enhancing its protective functions or inhibiting its pro-tumourigenic effects holds promise for improving cancer therapies. It is believed to influence immune evasion, angiogenesis, and tumour proliferation by modulating cytokine networks and oxidative stress responses. The interaction between haptoglobin and the immune system is particularly interesting in leukaemia, where immune suppression and altered inflammatory responses contribute to disease progression (Schaer et al., 2014). Additionally, changes in haptoglobin expression in malignant conditions can affect iron metabolism and erythropoiesis, further complicating disease pathology (Dobryszycka, 1997).

The necessity for a drug candidate that binds to haptoglobin arises from its pivotal role in haemoglobin clearance, immune regulation, and inflammatory modulation. Targeting haptoglobin could offer therapeutic benefits in conditions characterised by excessive haemolysis, such as sickle cell disease, haemolytic anaemias, and autoimmune haemolytic conditions (Bulters et al., 2018). In these disorders, the overwhelming release of free haemoglobin exceeds the binding capacity of endogenous haptoglobin, leading to oxidative stress and endothelial dysfunction (Gáll et al., 2019). A drug stabilising or enhancing haptoglobin function could improve clearance and reduce oxidative damage. Additionally, modulating haptoglobin activity has potential therapeutic implications in inflammatory and immune-mediated diseases. Given its influence on cytokine networks, a targeted therapeutic could be designed to either enhance or inhibit specific haptoglobin interactions, offering new treatment strategies for conditions such as sepsis, chronic inflammatory diseases, and even cancer (Telen et al., 2019). Furthermore, haptoglobin-targeted therapies could play a role in cardiovascular diseases, where oxidative stress and inflammation contribute to atherosclerosis and thrombosis. Developing haptoglobin-targeting drugs requires a deep understanding of its structural and functional properties. Small molecules, peptides, or monoclonal antibodies that bind to haptoglobin could be designed to modulate its interactions with haemoglobin, immune receptors, or coagulation factors (Mantovani et al., 2022). The potential to engineer haptoglobin-mimetic molecules or recombinant haptoglobin variants with enhanced haemoglobin-binding capacity is an exciting avenue for therapeutic development. Understanding haptoglobin polymorphisms and their influence on drug efficacy will also be crucial in developing personalised therapeutic strategies (Ciccolini et al., 2011). Given its relevance in multiple physiological and pathological processes, haptoglobin represents a promising target for future drug discovery efforts, with implications spanning haematology, immunology, oncology, and cardiovascular medicine (Dunphy et al., 2021). By leveraging advances in structural biology, bioinformatics, and AI-driven drug design, novel therapeutic approaches targeting haptoglobin could offer significant clinical benefits in managing haemolytic, inflammatory, and malignant disorders—currently, no widely approved therapeutic drugs precisely target haptoglobin (Hp) for clinical use (Delanghe et al., 2024). Moreover, while haptoglobin is being explored as a therapeutic target in conditions such as haemolytic anaemias, sepsis, and cardiovascular diseases, there is still insufficient understanding of how specific modulation of haptoglobin activity could be leveraged to improve clinical outcomes. The challenge lies in designing therapies that selectively target haptoglobin’s pathological roles, such as its involvement in immune modulation and oxidative stress, without affecting its crucial physiological functions in haemoglobin scavenging and iron homeostasis. This gap in knowledge underscores the need for more refined drug discovery approaches and deeper mechanistic insights into haptoglobin’s diverse biological roles. Despite the promising therapeutic potential of haptoglobin modulation in various pathological conditions, a significant knowledge gap remains in understanding the precise molecular mechanisms through which haptoglobin influences disease progression. While its role in haemoglobin scavenging, immune regulation, and inflammation is well-documented, the development of specific, targeted therapies remains challenging due to a lack of detailed mechanistic insights into how haptoglobin’s interactions with other molecules can be modulated for therapeutic purposes. Additionally, the variation in haptoglobin phenotypic forms complicates the design of universal therapeutic agents, necessitating a more personalised approach to drug development. As such, further research is needed to unravel the exact role of haptoglobin in disease states and to identify effective strategies for targeting haptoglobin specifically without disrupting its critical physiological functions. Computational techniques, such as molecular docking and simulation studies, offer promising avenues to bridge this knowledge gap by enabling a systematic exploration of potential haptoglobin-targeting compounds. Integrating structural data, ligand interaction profiles and pharmacokinetic modelling provides valuable insights into protein-ligand complexes’ binding dynamics and stability, essential for identifying viable drug candidates. As demonstrated in this study, such computational workflows can expedite the identification of haptoglobin inhibitors, paving the way for experimental validation and, ultimately, the development of novel therapeutic agents. In Japan, plasma-purified haptoglobin has been utilised since 1985 to protect the kidneys from haemoglobin-induced toxicity in conditions like extracorporeal circulation, massive transfusion, and thermal injury (Wang and Bellomo, 2017). Additionally, haptoglobin-conjugated nanoparticles and liposomes are under development for targeted drug delivery to inflamed tissues or cancer sites, aiming to maximise therapeutic efficacy while minimising systemic adverse effects (Aram et al., 2022). Furthermore, by fine-tuning immune responses, monoclonal antibodies that modulate haptoglobin function are being investigated as potential treatments for inflammatory and autoimmune diseases. These developments suggest a growing interest in harnessing haptoglobin pathways for therapeutic purposes, although such interventions are not yet standard clinical practice (He et al., 2022).

In this study, we performed the molecular docking of the complete DrugBank library against human haptoglobin and identified some crucial drug candidates. The candidates were then taken for the interaction fingerprints to determine the interaction pattern. We also performed the study’s DFT and pharmacokinetics to understand the identified compounds better. The study was extended to the WaterMap, MD simulation and MM\GBSA to better understand the protein-ligand complexes’ suitability and stability.

## 2 Methods

**FIGURE 1:**
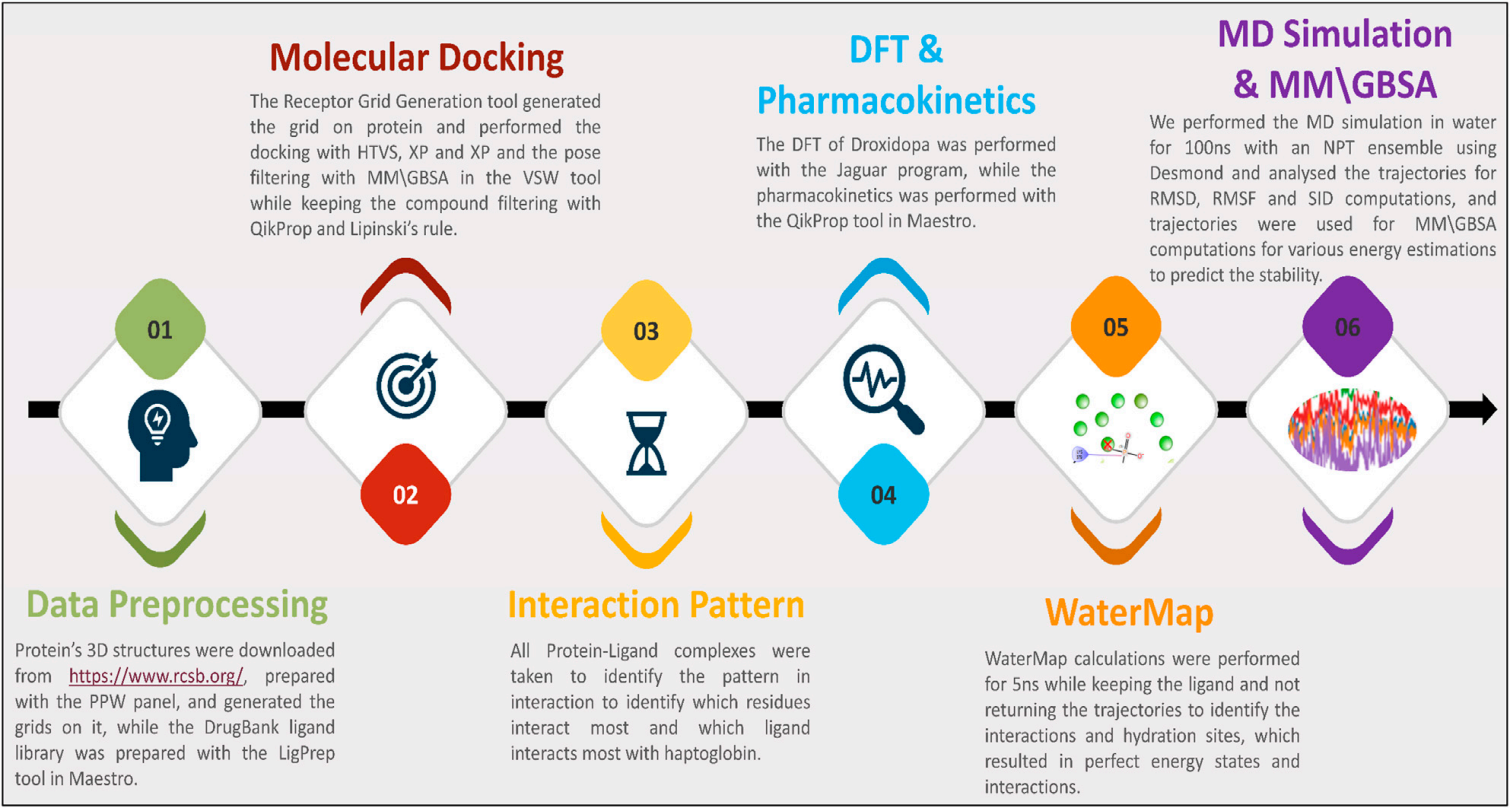
The study’s workflow shows data collection to identify potent drug candidates and validate them with WaterMap, MD Simulation, and binding free energy computations.

In this study, we have performed several studies on data collection, processing, docking, interaction fingerprints, DFT, pharmacokinetics, WaterMap, MD Simulation, binding free energy computations, and many more. We have plotted the workflow in Figure 1 to make it clearer. Further, the detailed methods are as follows-

### 2.1 Protein and ligand data collection and preparation

Protein preparation before docking is a crucial step to ensure accurate molecular interactions. It involves retrieving the protein structure from databases like PDB, removing water molecules, adding missing hydrogen atoms, optimising bond orders, and correcting protonation states based on physiological pH. Energy minimisation is performed to relieve steric clashes and stabilise the structure. Ligands, cofactors, and non-essential molecules are removed unless required for docking. Finally, the prepared protein is saved in an appropriate format for molecular docking simulations (Ahmad et al., 2023a; Famuyiwa et al., 2023; Ahmad et al., 2024b; Ahmad et al., 2025a). The structure of the human haptoglobin-haemoglobin complex (PDB ID: 4X0L) protein was downloaded from the RCSB-PDB database (https://www.rcsb.org/) that has chain A, B, and C of the Protein, Ligands, Solvents, metals/ions, and other heteroatoms, and prepared using the protein preparation workflow (PPW) in the Schrödinger’s Maestro (v.2022-2) (Maestro, 2022; Release, 2022f; Ahmad et al., 2025b). In the PPW, we capped the termini, filled in the missing side chains, reassigned all bond orders, created disulphide and zero bond orders to metals, filled in the loops using Prime, and generated the het states using Epik at pH 7.4 ± 2 (Jacobson et al., 2004; Shelley et al., 2007; Maestro, 2022). We further optimised the H-bond by sampling water orientation, using crystal symmetry, minimising the hydrogen of altered species, and using PROPKA (Olsson et al., 2011; Maestro, 2022). For the minimisation, we used the OPLS4 forcefield, all atoms to max 0.30Å RMSD and deleted water beyond 5Å to het-atoms after preparation, and we kept only the longest chain of the protein, which is chain C that was further used for the generating the grid (Jorgensen and Tirado-Rives, 1988; Maestro, 2022).

Ligand data collection and preparation involve retrieving ligand structures from databases like PubChem, ChEMBL, DrugBank, or ZINC or designing novel molecules using cheminformatics tools. The structures are optimised by assigning proper bond orders, tautomeric states, and stereochemistry. Energy minimisation uses force fields like MMFF94 or AMBER to achieve a stable conformation (Ahmad et al., 2022a; Ahmad et al., 2023b; Ahmad et al., 2024a). Protonation states are adjusted based on physiological pH, and partial atomic charges are assigned. Finally, the ligand is converted into a docking-compatible format (Ahmad et al., 2022b; Ahmad et al., 2023c; Ahmad and Raza, 2023). We downloaded the DrugBank ligand database for this study and prepared them with the LigPrep tool in Schrödinger’s Maestro (v.2022-2) (Maestro, 2022; Release, 2022d). In LigPrep, we kept filtering the compounds with more than 500 atoms, used OPLS4 force field and ionisation to generate the possible states at target pH 7 ± 2, and utilised the Epik classic and Desalt to generate tautomers (Jorgensen and Tirado-Rives, 1988; Shelley et al., 2007; Maestro, 2022). The stereoisomers computations were kept retaining the specified chiralities and generating at most 32 per compound, and the output file was written in SDF format for further use. The prepared ligand library was further used for direct docking as it has proper coordinates.

### 2.2 Molecular docking and pose processing studies

Receptor grid generation in docking defines the binding site for ligand placement and scoring. It involves selecting the target protein’s active site based on co-crystallised ligands, known functional residues, or blind docking predictions. Grid dimensions are optimised to encapsulate key interaction regions while avoiding excessive flexibility loss. Force fields like OPLS, AMBER, or CHARMM refine grid parameters, ensuring accurate electrostatic and van der Waals potential mapping (Jorgensen and Tirado-Rives, 1988; Al Khzem et al., 2024; Maestro, 2022). Properly generated grids enhance docking precision by restricting ligand sampling to relevant binding pockets (Ban et al., 2018; Ahmad et al., 2024c). In this study, we generate the grid on complete protein for blind docking using the Receptor Grid Generation tool in Schrödinger’s Maestro (v.2022-2), where we kept the scaling factor of one and partial cutoff of 0.25 (Maestro, 2022; 2022e). In the site tab, we kept displaying the box, specified ‘all’ residues to generate the grid on the centroid of the protein, adjusted the box to fit properly on the protein, and kept all advanced settings without disturbing it (Maestro, 2022; Release, 2022e). Molecular docking studies are pivotal in identifying new drug candidates by predicting the binding affinity and interaction patterns between small molecules and target proteins. The process begins with protein and ligand preparation, followed by grid generation to define the binding site. Docking algorithms, such as AutoDock, Glide, or GOLD, explore ligand conformations and binding modes within the active site. Scoring functions rank the docked poses based on binding energy and intermolecular interactions, including hydrogen bonding, hydrophobic contacts, and electrostatic forces. High-affinity candidates undergo further refinement through molecular dynamics simulations and binding free energy calculations to validate stability and specificity. The top-ranked compounds are shortlisted for *in vitro* and *in vivo* validation, bridging computational predictions with experimental drug discovery efforts. For the docking studies, we used the Glide in Schrödinger’s Maestro (v.2022-2), where we docked the compounds in multiple phases to save computational cost using the Virtual Screening Workflow (VSW) (Maestro, 2022; Release, 2022b). In the VSW panel, we browsed the prepared ligand library and kept generating unique properties of each compound and filtered them using the QikProp and Lipinski’s rule, skipped the preparation panel and browsed the protein’s generate grid in the receptor tab (Maestro, 2022; QikProp, 2022). We kept using Epik state penalties for docking in the docking panel and wrote interaction scores for residues within 12Å of the grid centre (Shelley et al., 2007; Maestro, 2022). The scaling factor was kept to 1, the partial charge cutoff was 0.15, and docking was done using high throughput virtual screening (HTVS), standard precision docking (SP), and extra precise docking (XP) (Maestro, 2022; Release, 2022b). In the HTVS, all 100% of compounds left after filtering were passed and after screening, 50% passed to SP and then screened and passed the top 50% to XP, where it was extensively docked while generating four poses per compound and 100% passed for the pose filtering with Prime-Molecular Mechanics Generalised Born Surface Area (MM\GBSA) (Al Khzem et al., 2025a). The complexes were further analysed and exported to CSV for further analysis, and the top five complexes were further analysed and taken for further study.

### 2.3 Interaction fingerprints computations

Molecular Interaction Fingerprints (MIFs) are computational descriptors that identify and analyse interaction patterns between ligands and target proteins in molecular docking and structure-based drug design. MIFs encode key molecular interactions such as hydrogen bonds, hydrophobic contacts, π-π stacking, salt bridges, and van der Waals forces into a compact numerical or binary format. By systematically comparing ligand binding profiles, MIFs help cluster, rank, and optimise drug candidates based on their similarity to known inhibitors’ interactions. They also assist in identifying conserved binding motifs across multiple docking poses or protein families (Tripathi et al., 2022; Al Khzem et al., 2025b). Advanced fingerprinting techniques, such as pharmacophore-based fingerprints and machine learning-driven MIF analysis, enhance drug discovery by predicting structure-activity relationships (SAR) and guiding lead optimisation. The MIF computations were performed using the Interaction Fingerprint tool in Schrödinger’s Maestro (v.2022-2), where we first selected the receptor-ligand complexes option and any contact types and did not perform the alignment as all protein cases were similar and generated the fingerprints without disturbing any advanced settings (Maestro, 2022). The interaction pattern was exported in a matrix plotted for further analysis. In the matrix, we plotted the count of interacting residues with names, the count of ligand interactions to identify which residues have better docking potential and chosen any contact types, and colour the main plot to determine the N to C terminal of the proteins and removed the non-interacting residues and displayed the docking scores for further analysis (Maestro, 2022).

### 2.4 Pharmacokinetics and density functional theory computations

Pharmacokinetics (PK) in drug design involves studying a drug candidate’s absorption, distribution, metabolism, and excretion (ADME) to assess its efficacy and safety. A well-optimised PK profile ensures the drug reaches its target at therapeutic concentrations without causing toxicity. Absorption determines how efficiently the drug enters systemic circulation, influenced by solubility and permeability. Depending on plasma protein binding and molecular properties, distribution assesses how the drug disperses in tissues (Lipinski, 2004; Famuyiwa et al., 2023). Metabolism, primarily occurring in the liver via cytochrome P450 enzymes, transforms the drug into active or inactive metabolites. Excretion through renal or biliary pathways determines drug clearance and half-life. Computational and experimental PK studies, including *in silico* ADMET predictions and *in vivo* pharmacokinetic assays, help optimise drug candidates by improving bioavailability, minimising off-target effects, and ensuring optimal dosing regimens. The pharmacokinetics profiling of all five identified compounds was performed using the QikProp tool in Schrödinger’s Maestro (v.2022-2) and kept Lipinski’s rule as a filter to have better screening, and these were further compared for all compounds with standard values of the tool (Chandrasekaran et al., 2018; Maestro, 2022; QikProp, 2022).

Density Functional Theory (DFT) is a quantum mechanical computational method used to study the electronic structure of molecules and materials. DFT plays a crucial role in drug design, optimising molecular geometries, calculating electronic properties, and predicting reactivity and intermolecular interactions. It provides insights into molecular orbital energies (HOMO-LUMO gap), charge distribution, dipole moments, and binding affinities, aiding in the rational design of potent drug candidates (Perepichka and Bryce, 2005). DFT is widely employed to understand ligand-protein interactions at the atomic level, helping predict binding modes and stability. It is instrumental in modelling covalent inhibitors, redox-active drugs, and metallodrugs by accurately describing electron density changes upon binding (Karwasra et al., 2022). The method also assists in calculating molecular electrostatic potential (MEP) maps, essential for identifying pharmacophoric regions. Based on system complexity, different functionals, such as B3LYP, M06–2X, and ωB97XD, and basis sets like 6-31G** and def2-TZVP. Despite its high accuracy, DFT can be computationally demanding for large biomolecular systems, requiring hybrid approaches like QM/MM (quantum mechanics/molecular mechanics) for efficient drug discovery applications. The DFT computations for all five complexes were performed using the Jaguar module in the Optimisation panel of Schrödinger’s Maestro (v.2022-2) (Bochevarov et al., 2013; Maestro, 2022; Release, 2022c). The compound was selected from the workspace, and the 6-31G basis set along with the Becke, 3-parameter, Lee-Yang-Parr with D3 dispersion correction (B3LYP-D3) theory was used for DFT (Witte et al., 2017; Maestro, 2022). The spin treatment was automatic, and Time-Dependent DFT (TDDFT) was performed for excited states with a grid density of medium. A three-body dispersion correction was applied with all applicable dispersion-corrected functionals. The Self-Consistent Field (SCF) accuracy level was set to “Quick,” with atomic overlap as the initial guess (Carbó and Riera, 2012; Maestro, 2022). The convergence criteria were maintained at 48 iterations, with an energy change threshold of 5 × 10^−5^ Hartree (Ha) and Root Mean Square (RMS) density matrix changes of 5 × 10^−6^. The Direct Inversion in the Iterative Subspace (DIIS) convergence scheme was applied (Hamilton and Pulay, 1986; Maestro, 2022). The optimisation was performed with a maximum of 100 steps, with a switch to analytic integrals near convergence and Schlegel Guess for the initial Hessian matrix (Schlegel, 2011; Maestro, 2022). Surfaces computations were carried out for electrostatic potential, average local ionisation energy, non-covalent interactions, electron density, spin density, and Molecular Orbitals (MO) for Highest Occupied Molecular Orbital (HOMO) to Lowest Unoccupied Molecular Orbital (LUMO) transitions, ranging from HOMO to LUMO+0 for Alpha and Beta states, as well as Natural Transition Orbitals (NTOs) for excited states (Perepichka and Bryce, 2005). The solvation model used was the Poisson-Boltzmann Finite element (PBF) solvent model, with water as the solvent, and optimisation was conducted in the gas phase. The output was written for proper analysis, and the Quantum Mechanics (QM) convergence monitor was used to analyse various energies for all five ligands.

### 2.5 WaterMap calculations

WaterMap is a computational method used to analyse the thermodynamic properties of water molecules in protein binding sites, playing a crucial role in rational drug design. It identifies and quantifies the energetics of water molecules in the active site by evaluating their free energy, entropy, and enthalpy contributions. Since water molecules mediate ligand-protein interactions, understanding their displacement and retention can optimise drug binding and potency (Kaczor et al., 2024). WaterMap simulations, based on MD simulation, categorise water molecules as either stable (structurally integral) or unstable (high-energy, displaceable). Displacing high-energy waters with hydrophobic ligand groups can enhance binding affinity while stabilising key water molecules can improve ligand specificity. This approach helps structure-based drug design (SBDD), particularly for optimising lead compounds, refining docking poses, and guiding fragment-based drug discovery. By providing insights into hydration energetics, WaterMap assists in designing drugs with improved solubility, bioavailability, and target selectivity, ultimately contributing to developing more effective therapeutics with optimised binding interactions (Ahmad and Raza, 2023; Kaczor et al., 2024). The WaterMap calculation was performed using the WaterMap-Perform Calculation panel in Schrödinger’s Maestro (v.2022-2), where we selected the ligand in the workspace and kept retaining the ligand and analysed the waters within 10Å of selected atoms (Bowers et al., 2006; Maestro, 2022; Release, 2022a). We kept truncating the protein for the simulation setup, using the OPLS4 force field and treating existing waters as solvents (Jorgensen and Tirado-Rives, 1988; Maestro, 2022). The simulation setup was kept to 5ns, did not return any trajectories, and kept the calculation job running on GPU. After the completion of the job, we used the WaterMap-examine results panel in Schrödinger’s Maestro (v.2022-2), where we went with the Analyse workspace option and then analysed the results where we computed various energies, including the enthalpy, entropy, free energy, overlap factor, distance and many other energy levels of the complexes (Bowers et al., 2006; Maestro, 2022). We exported the CSVs for further analysis and ligand interaction diagrams in 2D and 3D for better interactions and hydration site estimations.

### 2.6 Molecular dynamics simulation and binding free energy computations

Molecular Dynamics (MD) simulation is a computational technique used to study the dynamic behaviour of biomolecules, such as proteins, DNA, and ligands, over time at an atomic level. In drug design, MD simulations help evaluate ligand-protein interactions, stability, binding affinity, and conformational changes under physiological conditions. By solving Newton’s equations of motion for each atom, MD simulations provide insights into biomolecular flexibility, hydration effects, and energy landscapes. MD simulations start with system preparation, including solvation, charge neutralisation, and force field assignment (e.g., CHARMM, AMBER, OPLS) (Soares et al., 2003). The system undergoes energy minimisation, equilibration (NVT/NPT ensembles), and production runs, typically ranging from nanoseconds to microseconds. Key analyses include root-mean-square deviation (RMSD) for structural stability, root-mean-square fluctuation (RMSF) for flexibility, hydrogen bonding, solvent-accessible surface area (SASA), and binding free energy calculations (MM-PBSA/MM-GBSA). MD simulations refine docking results by confirming ligand stability, identifying water-mediated interactions, and predicting allosteric binding sites. They also aid in lead optimisation, drug resistance studies, and personalised drug design by modelling mutations and drug-target adaptations, making them essential in modern computational drug discovery. In this study, we performed the MD Simulation using Desmond Package in Schrödinger’s Maestro (v.2022-2) available from https://www.deshawresearch.com/resources.html, which conducts the whole analysis in three parts starting from building the system file, production run and analysing the trajectories (Bowers et al., 2006; Maestro, 2022; Release, 2022a). The system builder tool in Schrödinger’s Maestro (v.2022-2) was used to prepare the system file where we used the predefined model TIP3P and boundary conditions in orthorhombic condition with 10 × 10 × 10 Å in buffer and showed the boundary box (Mark and Nilsson, 2001; Maestro, 2022). The ion and salt placement were excluded with 20 Å and neutralises the complexes by adding 0Na^+^, 0 Na^+^, 1Na^+^, 2Na^+^, 0Na^+^, 0Na^+^ in haptoglobin in complex with L-histidinol phosphate (DB03997), L-Gluconic Acid (DB04304), 4-bromo-3-(carboxymethoxy)-5-(4-hydroxyphenyl)thiophene-2-carboxylic acid (DB07197), 3-O-Methylfructose (DB02438), and Glutamine hydroxamate (DB02446), and uses the OPLS4 forcefield and kept the job running to prepare the system file to run the MD Simulation jobs (Jorgensen and Tirado-Rives, 1988; Maestro, 2022). The production jobs were kept for the 100ns after loading the complex file, where we kept the recording interval of 100 ps, which recorded a total of 1,000 frames per complex. The NPT ensemble class was kept at 300 K temperature and 1.01325 bar pressure, and the model was relaxed before the production run (McDonald, 1972; Maestro, 2022). Further, we also kept running the analysis job, which generated the. eaf file for analysis with the Simulation Interaction Diagram tool used for exporting the figures and data for further detailed analysis (Bowers et al., 2006; Maestro, 2022).

Molecular Mechanics Generalised Born Surface Area (MM\GBSA) is a widely used end-point free energy calculation method applied to MD simulation trajectories for evaluating ligand binding affinities. It balances accuracy and computational efficiency, making it a preferred approach for refining docking results and validating ligand-protein interactions. In MM\GBSA, the total binding free energy (ΔGbind) is computed as the difference between the free energy of the complex and the sum of the free energies of the unbound protein and ligand. In MM\GBSA, the total binding free energy (ΔG_bind_​) is computed as:

ΔG=bindG complex‐ (G protein+G)ligand

where G represents the free energy of each molecular state. The total free energy is derived from the sum of molecular mechanics energy, which includes bonded, electrostatic, and van der Waals interactions, solvation-free energy contributed by the polar component from the Generalised Born (GB) model and the non-polar component from solvent-accessible surface area (SASA), and optionally, the entropic contribution. Following an MD simulation, MM\GBSA is applied to extract snapshots from the equilibrated phase of the trajectory, typically using molecular dynamics engines like AMBER, GROMACS, or Schrödinger’s Prime MM\GBSA module (Yadav et al., 2022; Maestro, 2022). The method is instrumental in refining docking results by incorporating solvation and entropic contributions, assessing ligand stability by evaluating retention in the binding pocket over time, and predicting binding affinity by providing scores that correlate well with experimental data. Additionally, MM\GBSA helps study the impact of protein mutations on drug binding, making it a valuable tool in drug resistance studies. When combined with MD simulations, MM\GBSA enhances the accuracy of computational drug discovery by offering detailed insights into ligand-protein binding energetics and guiding the selection and optimisation of potent drug candidates. We used a custom python script (mmgbsa.py) to run the MM\GBSA on all five complexes’ trajectories in Schrödinger’s Maestro (v.2022-2) (Tuccinardi, 2021; Maestro, 2022) using the bash script where first we called the Schrödinger run in the environment and then executed the python file by the following commands-

export SCHRODINGER=/opt/Schrödinger‐2022‐2/ SCHRODINGER/run thermal_mmgbsa.py desmond_md_job_NAME‐out.cms

After MM\GBSA jobs, we analysed various energies, including the binding free energy and total complex energy and plotted the Figure to make energies clear for 0 to 1,000 frames (Wang et al., 2019; Tuccinardi, 2021; Maestro, 2022).

## 3 Results

The molecular docking studies have resulted in many promising compounds, however, after careful analysis, we identified L-histidinol phosphate (DB03997), L-Gluconic Acid (DB04304), 4-bromo-3-(carboxymethoxy)-5-(4-hydroxyphenyl)thiophene-2-carboxylic acid (DB07197), 3-O-Methylfructose (DB02438), and Glutamine hydroxamate (DB02446) as the best compounds for haptoglobin that further were validated with many analysis. The detailed analysis for each step is as follows-

### 3.1 Protein structure analysis and validation

The protein preparation results from the Schrödinger Maestro PPW provide a comprehensive analysis of the energetic parameters associated with the system, offering critical insights into its structural stability, molecular interactions, and force field contributions. The system’s total energy was recorded at −3.52993 × 10^3^ kcal/mol, representing the molecular system’s combined potential and kinetic energy contributions. Since the total kinetic energy was 0.00000 kcal/mol, indicating the system was in a minimised state without dynamic motion, as expected during the initial stages of structural refinement. The total potential energy, also recorded as −3.52993 × 10^3^ kcal/mol, reflects the sum of all molecular interactions contributing to the stability of the prepared protein structure. The system’s temperature was noted as 0.000 K, indicating that the energy minimisation process was performed at a static state without the influence of thermal fluctuations, ensuring that the calculations reflected a fully optimised structure under a vacuum or implicit solvent model. The bond stretch energy was calculated at 262.462 kcal/mol among the bonded interaction components, reflecting the energy required to maintain bond lengths near their equilibrium values as defined by the applied force field. At 1.21983 × 10^3^ kcal/mol, the angle bending energy represents the strain energy associated with deviations from ideal bond angles. The torsion angle energy was computed as 861.749 kcal/mol, accounting for the rotational strain energy within flexible dihedral angles of the protein, which is critical for evaluating the conformational preferences of secondary structure elements. Interestingly, the restraining energy for torsions was 0.00000 kcal/mol, signifying that no additional constraints were imposed on dihedral angles, allowing for natural conformational flexibility. Non-bonded interactions are crucial in determining the protein’s stability, particularly van der Waals (Lennard-Jones) interactions and electrostatic contributions. The 1,4 Lennard-Jones energy, which quantifies the non-bonded steric interactions between atoms separated by three covalent bonds, was 2.49605 × 10^3^ kcal/mol, indicating significant steric repulsion and attraction forces within short-range molecular contacts. The 1,4 electrostatic energy, computed as 1.18138 × 10^3^ kcal/mol, reflects the Coulombic interactions between atoms separated by three bonds, influencing the protein’s dipole alignment and local charge distribution. The Lennard-Jones energy, representing overall van der Waals interactions across the entire system, was −5.81707 × 10^3^ kcal/mol, with the large negative value indicating a strong stabilising effect due to favourable dispersion forces between non-bonded atoms. Similarly, the electrostatic energy was −3.76839 × 10^3^ kcal/mol, which accounts for the long-range Coulombic interactions and charge-charge stabilisation effects within the protein. The hydrogen bond energy, recorded at 0.00000 kcal/mol, suggests that explicit hydrogen bonding interactions were either negligible or implicitly incorporated within the force field’s electrostatic treatment, which may depend on the solvation model or minimisation settings. These energy components provide a detailed assessment of the prepared protein’s structural integrity and interaction potential. The significant contributions from angle bending and torsion energy highlight the importance of proper dihedral conformations, while the dominant negative electrostatic and Lennard-Jones energies confirm that the protein structure is well-optimised for molecular docking or further computational studies. These parameters collectively ensure the protein is in a low-energy, stable state, suitable for downstream applications such as molecular docking, molecular dynamics simulations, or ligand binding studies.

**FIGURE 2:**
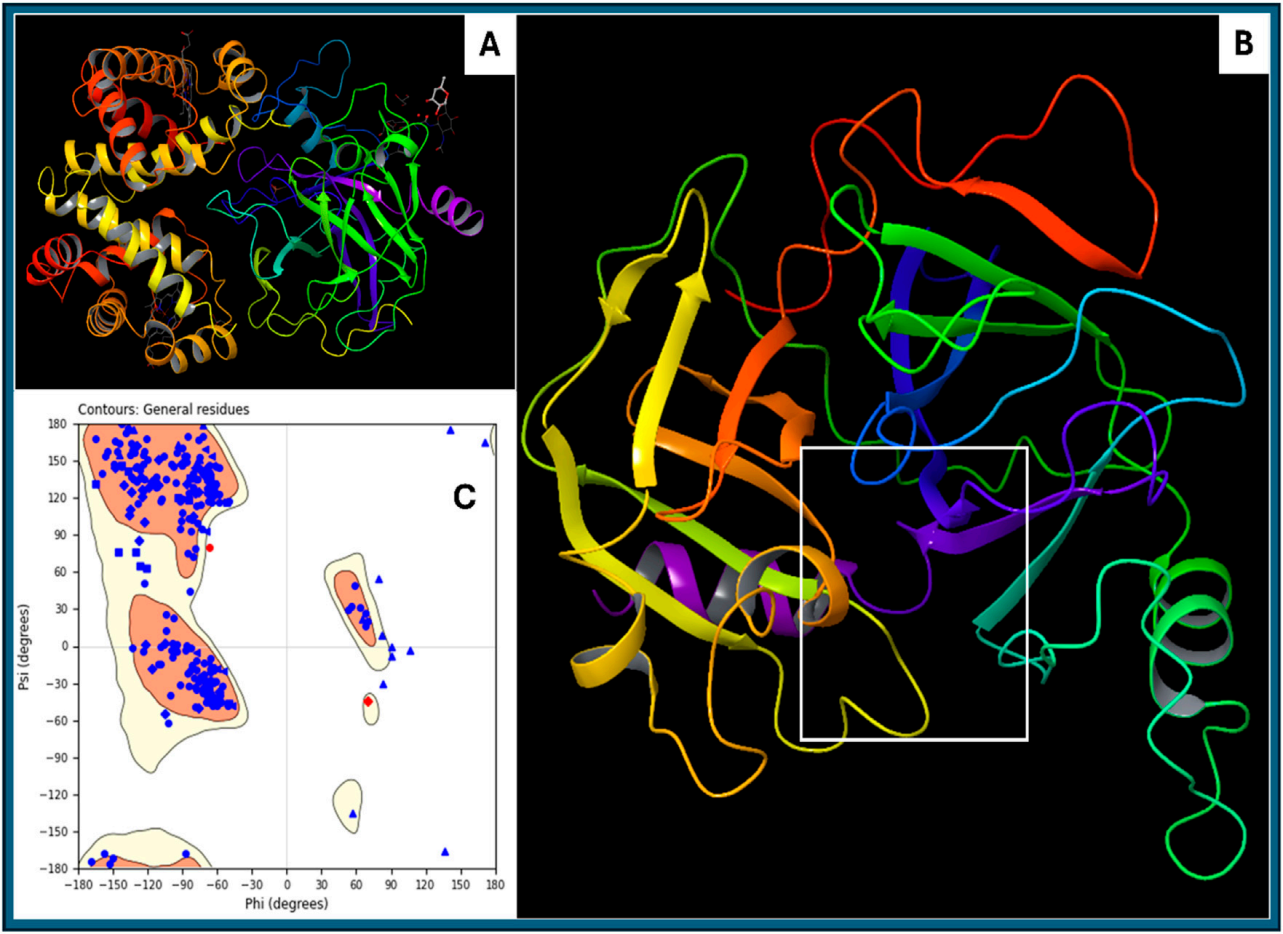
Showing the **(A)** downloaded haptoglobin structure in 3D, **(B)** prepared clean structure of haptoglobin with marked ligand binding site (white box), and **(C)** Ramachandran Plot for the Prepared structure of the protein.

The protein descriptor analysis for haptoglobin provides a detailed evaluation of its physicochemical, structural, and energetic properties. Aggregation propensity is captured through AGGRESCAN_Nr_hotspots (7) and AGGRESCAN_a3v_value (0.003574713), indicating a relatively low aggregation tendency. The amino acid composition (Aa_Composition: 1,479.6) and comparison with SwissProt (1,488.49) reveal the general residue distribution within the protein and its alignment with standard protein databases. Amino acid flexibility (Aa_Flexibility_VTR: 259.508) provides insights into the conformational adaptability of the protein backbone. Hydrophobicity and solvent accessibility parameters are essential for understanding protein stability and interactions. The total solvent-accessible surface area (All_SASA: 12,215.727 Å^2^) indicates the overall exposure of residues to the solvent. The hydrophobic surface areas (All_Hydrophobic_SASA: 502.119 Å^2^, All_Hydrophilic_SASA: 0.45075598 Å^2^) highlight the balance between hydrophilic and hydrophobic interactions that influence protein folding and stability. The hydrophobic patch energy (All_Hydrophobic_Patch_Energy: 397.444 kcal/mol) quantifies the stabilisation effect of hydrophobic residues, while positive and negative patch energies (All_Positive_Patch_Energy: 3,496.422 kcal/mol, All_Negative_Patch_Energy: 3,012.522 kcal/mol) illustrate the distribution of charge patches across the protein surface. Electrostatic properties play a critical role in protein function and interactions. The dipole moment (All_Dipole_Moment: 554.217 Debye) signifies the charge distribution asymmetry, influencing molecular recognition. The formal charge (All_Formal_Charge: 7.05E-13 eV) indicates near-neutrality, while the apparent charge (Apparent_Charge_eV: 2.39E-13 eV) suggests minimal net charge effects in physiological conditions. The zeta potential (All_Zeta_Potential: 2.38E-12 mV) gives insights into colloidal stability and aggregation tendencies. Secondary structure propensities provide insights into the folding characteristics of haptoglobin. The alpha-helix content, as predicted by Chou-Fasman (259.82), Deleage-Roux (260.787), and Levitt (263.52), indicates a stable helical structure. Similarly, beta-sheet content (Chou-Fasman: 264.6, Deleage-Roux: 261.625, Levitt: 258.86) reveals a well-balanced sheet composition. Beta-turn tendencies (252.75–252.82) and coil content (Coil_Deleage_Roux: 257.664) further support the structural complexity. Protein compactness and shape are inferred from the moment of inertia values (Inertia_X: 0.902, Inertia_Y: 0.930, Inertia_Z: 1.115) and radius of gyration (33.06 Å), which indicate a relatively compact structure. Atomic contact energy (−477.181 kcal/mol) suggests favourable intra-protein interactions, crucial for maintaining structural integrity. The hydrophobicity indices (Kyte-Doolittle: −77.6, Janin: −36.6, Eisenberg: 11.26) confirm that haptoglobin exhibits a moderately hydrophilic nature. Charge-based properties include total negative SASA (Total_negative_SASA: 1,491.4 Å^2^) and total positive SASA (Total_positive_SASA: 1,574.1 Å^2^), which indicate a balanced distribution of charged residues. The net charge (Net_Charge_model_based: 2.32, Net_Charge_propka_based: 1.20) suggests a slight positive charge under physiological conditions, which could impact its interaction with negatively charged molecules. Other key descriptors include molecular weight (33,366 Da), refractivity (22,658), and sedimentation coefficient (2.16E-13 Svedbergs), providing insights into protein mass, optical properties, and potential behaviour in solution. The hydrophobic patch size (Avg_Size_Hyd_Patches: 143.01, Max_Size_Hyd_Patches: 379.72) quantifies hydrophobic clustering, which is relevant for ligand binding and self-assembly. This comprehensive descriptor analysis confirms that haptoglobin is a structurally stable, hydrophilic protein with well-balanced secondary structure elements, moderate aggregation propensity, and defined charge distribution. These properties are crucial for understanding its biological function, interactions, and potential modifications for biotechnological or therapeutic applications. Further, in Figure 2, we show the original downloaded structure and prepared clean structures with ligand binding site in 3D format, as well as Ramachandran Plot for the prepared protein to clarify the structural understanding.

### 3.2 Molecular docking analysis

The molecular docking study Haptoglobin protein (PDB ID: 4X0L) was performed, revealing several promising results. However, five of the best ligands were selected for a detailed study. The docking results provide insights into the binding affinities, hydrogen bonding interactions, salt bridges, and overall stability of the ligand-protein complexes. The docking simulation of haptoglobin with L-histidinol phosphate resulted in a docking score of −7.96 kcal/mol and an MMGBSA binding free energy of −26.23 kcal/mol. The ligand formed two hydrogen bonds with ASN203 and ASP378 residues through its N + H3 atom. Additionally, two salt bridges were established between the ASP378 residue and the N + H3 atom and between the LYS378 residue and the phosphorus atom of L-histidinol phosphate. The high negative binding energy indicates a strong and stable interaction. L-gluconic acid exhibited a docking score of −7.74 kcal/mol and an MMGBSA binding free energy of −19.09 kcal/mol. The ligand formed five hydrogen bonds with ASN203, ASP378, and LYS378 residues via two OH atoms, while the ARG286 residue interacted with the ligand through an oxygen atom. A salt bridge interaction was also observed between the ARG286 residue and the oxygen atom of L-Gluconic acid. The multiple hydrogen bonds contribute significantly to the ligand’s stability within the binding site. Haptoglobin Complex with 4-Bromo-3-(Carboxymethoxy)-5-(4-Hydroxyphenyl) Thiophene-2-Carboxylic Acid (DB07197) exhibited a docking score of −6.92 kcal/mol and an MMGBSA binding free energy of −4.12 kcal/mol. Hydrogen bond interactions were established between THR200, SER376, and ARG286 residues with the OH and O atoms of the ligand. Additionally, a π-cation interaction was observed between the benzene ring and LYS379 residue, and a salt bridge was formed between ARG286 and the oxygen atom of the ligand. A π-cation interaction suggests possible stabilisation through electrostatic and hydrophobic interactions. The docking analysis of haptoglobin with 3-O-Methylfructose in Linear Form showed a docking score of −6.16 kcal/mol and an MMGBSA binding free energy of −10.97 kcal/mol. This ligand formed five hydrogen bonds with THR200, ASN203, and ASP378 residues via three OH atoms and LYS379 residue through the oxygen atom of the ligand. The relatively lower docking score and binding energy suggest moderate interaction strength with the protein. Glutamine hydroxamate exhibited the lowest docking score of −5.57 kcal/mol and an MMGBSA binding free energy of −1.00 kcal/mol, indicating a weak binding affinity. The ligand formed four hydrogen bonds, involving ASN203 and ASP378 residues with the N + H3 atom, ASP378 residue with the NH atom, and THR200 and SER376 residues with the OH atom. Two salt bridges were also formed between ASP378 and LYS379 residues with the ligand’s N + H3 atom and oxygen atom. The weak binding energy suggests this ligand may not have a strong therapeutic potential for haptoglobin targeting.

**FIGURE 3:**
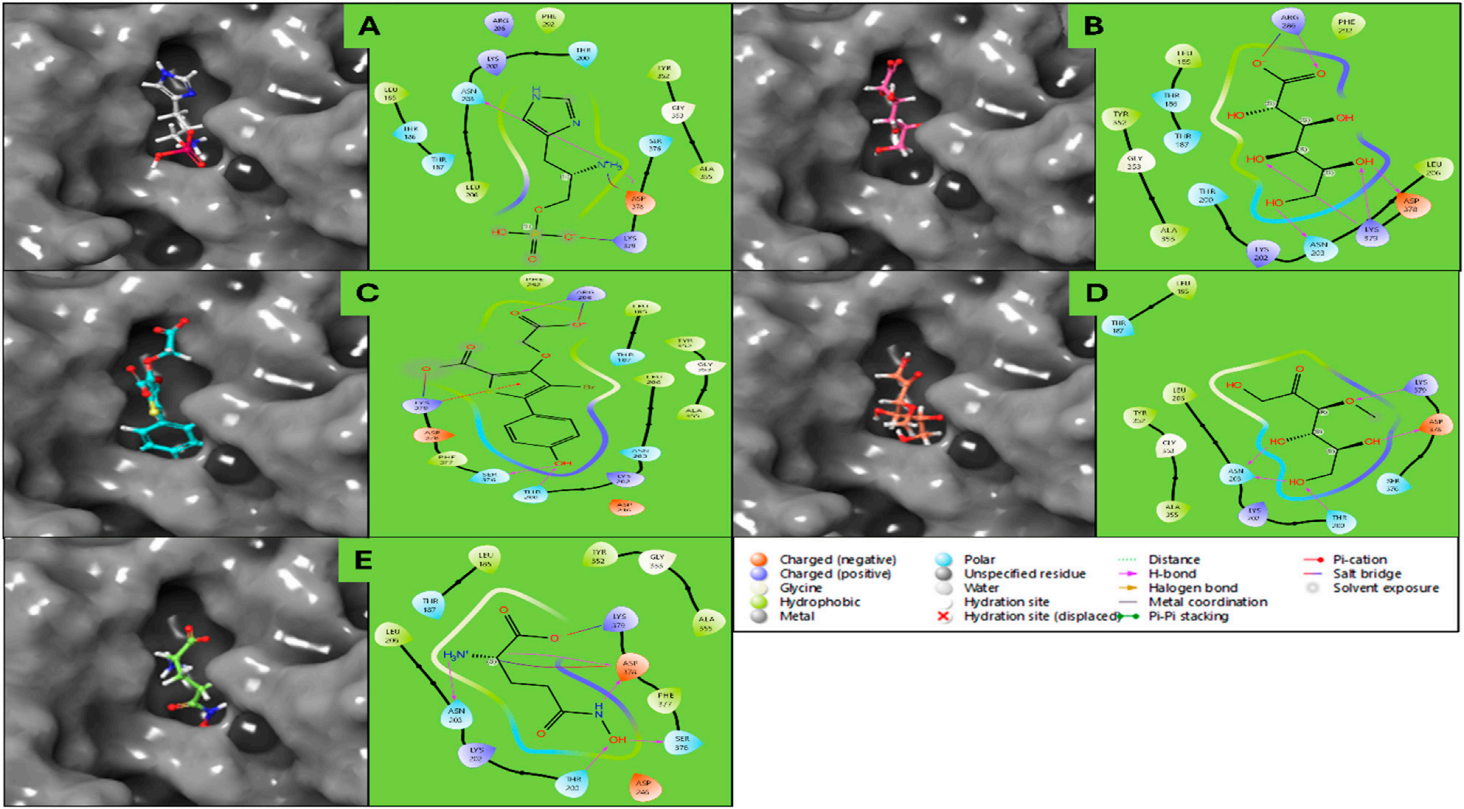
Showing the Docking Poses in 3D and 2D for haptoglobin in complex with **(A)** L-histidinol phosphate (DB03997), **(B)** L-Gluconic Acid (DB04304), **(C)** 4-bromo-3-(carboxymethoxy)-5-(4-hydroxyphenyl)thiophene-2-carboxylic acid (DB07197), **(D)** 3-O-Methylfructose (DB02438), and **(E)** Glutamine hydroxamate (DB02446), and the legend is provided to make the bond and residues types more clear.

The docking analysis of human haptoglobin (PDB ID: 4X0L) with five different ligands provides insights into their binding affinities, hydrogen bond interactions, and stability within the protein’s active site. Among the tested ligands, L-Histidinol Phosphate (DB03997) demonstrated the highest docking score (−7.96 kcal/mol) and the most stable binding free energy (−26.23 kcal/mol), suggesting strong binding interactions facilitated by hydrogen bonds and salt bridges. Similarly, L-Gluconic Acid (DB04304) exhibited a relatively high docking score (−7.74 kcal/mol) and significant stability (−19.09 kcal/mol), owing to multiple hydrogen bonds and a salt bridge with ARG286. These results suggest that these ligands could be promising candidates for haptoglobin-targeted applications. Conversely, Glutamine Hydroxamate (DB02446) showed the weakest docking score (−5.57 kcal/mol) and the least favourable MMGBSA binding free energy (−1.00 kcal/mol), indicating poor binding stability. The moderate binding of 3-O-Methylfructose (DB02438) and 4-Bromo-3-(Carboxymethoxy)-5-(4-Hydroxyphenyl) Thiophene-2-Carboxylic Acid (DB07197) suggests that while they form multiple interactions, their overall binding strengths are not as robust as L-Histidinol Phosphate or L-Gluconic Acid. Hydrogen bonding played a crucial role in stabilising the ligand-protein interactions, with ASN203, ASP378, and LYS378 emerging as key residues across multiple ligands. Additionally, salt bridges involving ASP378 and ARG286 were observed in several cases, contributing to enhanced ligand binding stability. A π-cation interaction in DB07197 with LYS379 indicates a potential stabilising force via electrostatic interactions. Further experimental validation, including molecular dynamics simulations and biochemical assays, would be necessary to confirm their potential as drug candidates for haptoglobin-related therapeutic applications. Furthermore, detailed results for each energy level are shown in Table 1 and Figure 3 for the 3D and 2D docking poses to clarify what bonds and residues are involved in the interactions.

**TABLE 1:** Showing the DrugBank IDs with docking scores, XP scores, MM\GBSA scores (all in Kcal/mol) and many other scores generated during the docking studies.

PDB ID	DrugBank IDs	State Penalty	Docking Score	XP GScore	XP HBond
4X0L	DB03997	2.11	−7.96	−10.07	−1.51
4X0L	DB04304	0.00	−7.74	−7.74	−3.73
4X0L	DB07197	0.00	−6.92	−6.92	−2.00
4X0L	DB02438	0.00	−6.16	−6.16	−2.81
4X0L	DB02446	0.00	−5.58	−5.58	−1.91

PDB ID	DrugBank IDs	MMGBSA dG Bind	Complex Energy	ligand efficiency sa	ligand efficiency ln
4X0L	DB03997	−26.23	−10371.73	−1.37	−2.19
4X0L	DB04304	−19.09	−10257.00	−1.40	−2.17
4X0L	DB07197	−4.12	−10307.71	−0.91	−1.71
4X0L	DB02438	−10.97	−10230.23	−1.11	−1.73
4X0L	DB02446	−1.00	−10300.29	−1.13	−1.64

### 3.3 Molecular interaction pattern analysis

**FIGURE 4:**
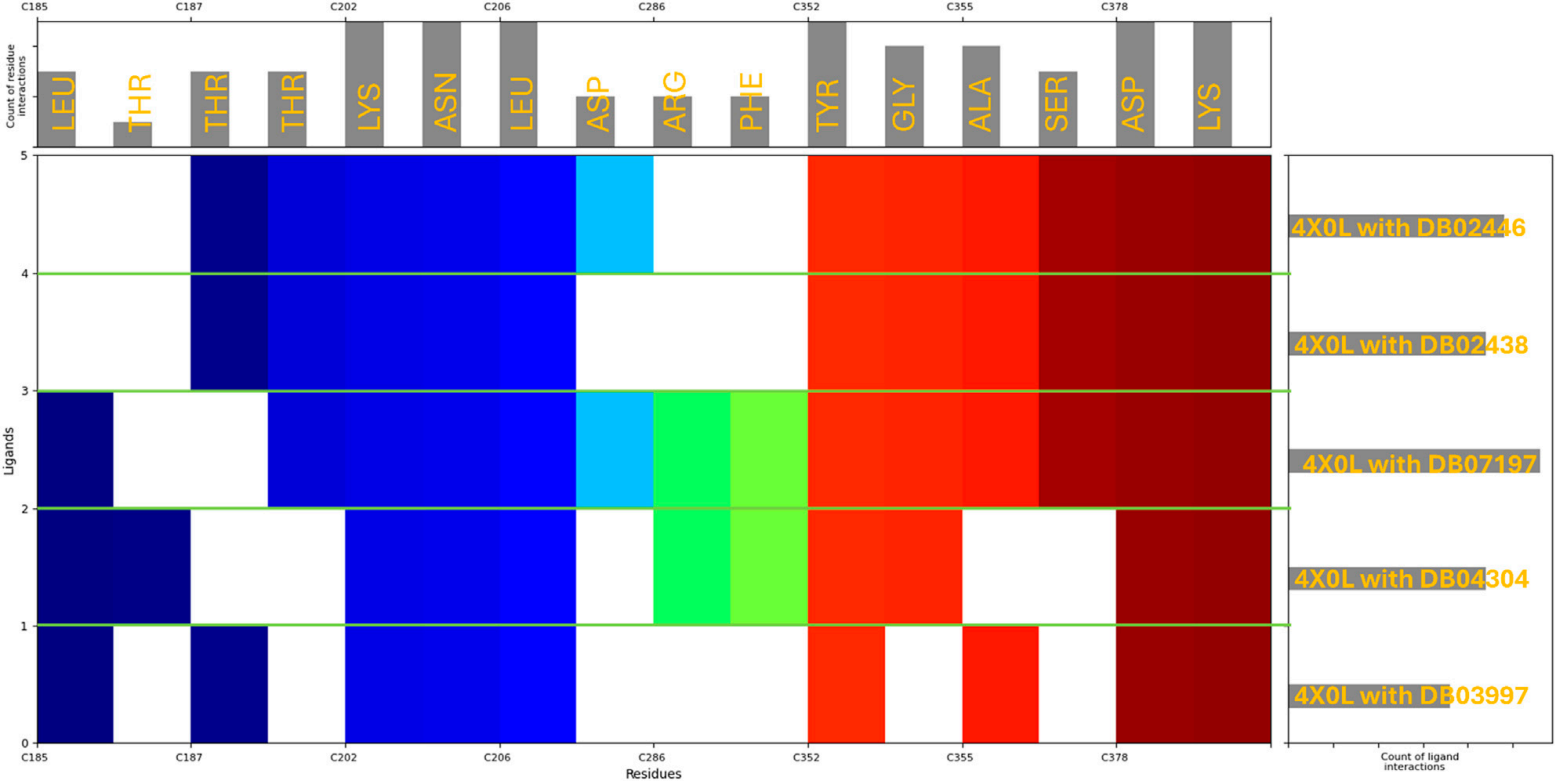
Showing the molecular interaction fingerprints of the docked poses of haptoglobin in complex with L-histidinol phosphate (DB03997), L-Gluconic Acid (DB04304), 4-bromo-3-(carboxymethoxy)-5-(4-hydroxyphenyl)thiophene-2-carboxylic acid (DB07197), 3-O-Methylfructose (DB02438), and Glutamine hydroxamate (DB02446). The upper bar shows the residues forming the interactions with its counts, and the right side bars show the count of ligand interaction with Haptoglobin, while the N to C terminal in the main plot is shown with different colours.

The molecular interaction fingerprint analysis of the docked poses revealed the key residues contributing to the binding of five ligands to human haptoglobin. The identified residues belong to diverse categories, including positively charged (basic), negatively charged (acidic), polar uncharged, non-polar (hydrophobic), and aromatic amino acids, each playing a distinct role in ligand recognition, stability, and affinity. Basic Residues during the interactions were–Lysine (LYS) (10 occurrences) and Arginine (ARG) (2 occurrences). Lysine (LYS), appearing in significant abundance (10 occurrences), highlights the strong electrostatic attraction between the protein and negatively charged or polar ligand groups. LYS contains a positively charged ε-amino group, which readily forms salt bridges and hydrogen bonds with carboxylate (-COO^-^) and phosphate (-PO_4_^2-^) groups from the ligands. This suggests that haptoglobin favours interactions with anionic ligands or those possessing electronegative atoms. The high frequency of lysine also implies its stabilising role by forming long-range electrostatic interactions that contribute to ligand retention. Arginine (ARG), present in two instances, plays a complementary role due to its guanidinium group, which can form strong bidentate hydrogen bonds and π-cation interactions with aromatic moieties of the ligands. Acidic Residues during the interactions were–Aspartic Acid (ASP) (7 occurrences). Aspartic acid, a negatively charged residue at physiological pH, frequently contributes to salt bridge formation with positively charged amine groups on the ligands. The significant occurrence of ASP residues (7 counts) highlights its role in stabilising ligands that contain positively charged nitrogen groups, such as protonated amines (–NH_3_^+^). ASP also participates in hydrogen bonding, further reinforcing ligand binding. Polar Uncharged Residues during the interactions were–Threonine (THR) (7 occurrences), Asparagine (ASN) (5 occurrences), and Serine (SER) (3 occurrences). Polar residues such as THR, ASN, and SER are heavily involved in hydrogen bond interactions, particularly with hydroxyl (-OH), amide (-CONH_2_), or carboxyl (-COOH) groups from the ligands. Threonine (THR) (7 occurrences) is often involved in hydroxyl-mediated hydrogen bonding, contributing to ligand orientation and specificity. Asparagine (ASN) (5 occurrences) plays a similar role, especially in interactions with backbone carbonyl groups. Serine (SER) (3 occurrences) can also establish side-chain hydrogen bonds, further enhancing ligand stability within the binding pocket. Non-polar (Hydrophobic) Residues during the interactions were–Leucine (LEU) (8 occurrences), Alanine (ALA) (4 occurrences), and Glycine (GLY) (4 occurrences). Hydrophobic residues such as LEU, ALA, and GLY contribute to hydrophobic pocket formation, which is crucial for ligands with non-polar moieties. Leucine (LEU), appearing 8 times, is a major hydrophobic interaction contributor, helping to create a favourable van der Waals environment for non-polar ligand regions. Alanine (ALA) and glycine (GLY) provide structural flexibility to the binding site, which may assist in accommodating different ligand conformations. Aromatic Residues during the interactions were–Tyrosine (TYR) (5 occurrences) and Phenylalanine (PHE) (2 occurrences). Aromatic residues such as TYR and PHE are essential for π–π stacking and hydrophobic interactions with planar and aromatic ligand structures. Tyrosine (TYR) (5 occurrences) can also engage in hydrogen bonding through its hydroxyl (-OH) group, making it versatile in ligand recognition. Phenylalanine (PHE) (2 occurrences) mainly contributes to hydrophobic stabilisation and π-interactions with ligand rings. The fingerprint analysis of haptoglobin-ligand complexes reveals that positively charged lysine residues (LYS) dominate ligand binding, suggesting a strong preference for interacting with negatively charged or polar ligands. Additionally, aspartic acid (ASP) residues frequently engage in electrostatic and hydrogen bonding interactions, highlighting their importance in stabilising positively charged ligand groups. Hydrophobic residues such as LEU, ALA, and PHE contribute to non-polar interactions, while TYR, THR, ASN, and SER residues play crucial roles in hydrogen bonding and ligand orientation, and in Figure 4, we have plotted the whole fingerprints. These findings provide valuable insights into the molecular determinants governing ligand affinity and specificity for haptoglobin, which can be leveraged in drug design and optimisation strategies.

### 3.4 Density functional theory and pharmacokinetics analysis

The Density Functional Theory (DFT) calculations for the five molecules, DB03997, DB04304, DB07197, DB02438, and DB02446, were performed using the B3LYP-D3 functional within the Jaguar program in Schrödinger, employing the 6-31G** basis set in a solvated environment (Bochevarov et al., 2013; Maestro, 2022). All molecules were treated as singlet states with a spin multiplicity of one. The optimisation calculations led to different convergence categories, with DB03997 reaching category 1, DB04304 at category 2, and both DB07197 and DB02438 converging at category 4. The number of canonical orbitals varied significantly among the molecules, ranging from 215 for DB02446 to 373 for DB07197, reflecting their differing electronic structures. The gas-phase energies spanned from −1,042.5006 au for DB03997 to −3,923.0454 au for DB07197, with the solution-phase energies exhibiting a similar trend, slightly lowered due to solvation effects, leading to solvation energies that were highest in magnitude for DB07197 (−198.37 kcal/mol), followed by DB04304 (−67.64 kcal/mol), DB03997 (−37.55 kcal/mol), DB02438 (−18.75 kcal/mol), and DB02446 (−17.00 kcal/mol). These variations indicate differing levels of stabilisation upon solvation. The Frontier molecular orbital energies provided insights into the electronic characteristics of these molecules. The highest occupied molecular orbital (HOMO) energy values ranged from −0.2604 au for DB02438 to −0.2140 au for DB07197, whereas the lowest unoccupied molecular orbital (LUMO) energies exhibited values between −0.0518 au for DB07197 and 0.0324 au for DB04304. The HOMO-LUMO gap, which signifies the chemical reactivity and stability, was the largest for DB04304 and the smallest for DB07197, indicating that DB07197 may be the most reactive among the studied molecules. The vibrational frequency analysis revealed that DB03997 and DB07197 exhibited negative frequencies of −102.19 cm^-1^ and -1,396.09 cm^-1^, respectively, suggesting possible saddle points or non-stationary configurations, whereas the other molecules had their lowest vibrational frequencies in positive values, indicating stable, optimised structures. The highest vibrational frequencies were consistent among all molecules, between 3,563.94 cm^-1^ and 3,731.21 cm^-1^. Thermodynamic properties calculated at 298.15 K and 1 atm further illustrated these molecules’ stability and entropy-driven behaviour. The zero-point energies ranged from 106.61 kcal/mol for DB02446 to 140.66 kcal/mol for DB02438, with entropy values spanning from 101.35 cal/mol/K for DB02446 to 126.44 cal/mol/K for DB07197, indicating that DB07197 possesses the most disordered state at room temperature. Enthalpy values were found to be the highest for DB07197 (10.57 kcal/mol) and the lowest for DB02446 (7.47 kcal/mol), whereas free energy values followed a similar trend, suggesting that DB07197 has the most favourable thermodynamic stability. Internal energy and heat capacity values showed a consistent pattern, with DB07197 exhibiting the highest heat capacity (62.95 cal/mol/K), indicative of greater molecular flexibility. The natural logarithm of the partition function (ln(Q)) was also the highest for DB07197 (45.79 kcal/mol), correlating with its increased conformational entropy. Electrostatic potential (ESP) analysis provided valuable insights into charge distribution, revealing that DB07197 exhibited the most negative ESP minimum (−238.72 kcal/mol) and a highly negative ESP mean (−135.33 kcal/mol), suggesting strong electron-withdrawing regions, whereas DB03997 had a more balanced ESP distribution with a mean value of 2.01 kcal/mol. The ESP balance values were nearly negligible for DB04304 and DB07197, indicating highly polarised charge distributions, whereas DB03997, DB02438, and DB02446 exhibited more balanced charge dispersion. The ESP local polarity was the highest for DB07197 (50.96 kcal/mol), implying that this molecule has the most localised charge separation. The average local ionisation energy values further reinforced these electronic characteristics. The minimum ALIE ranged from 196.85 kcal/mol for DB03997 to 230.56 kcal/mol for DB02438, whereas.

**FIGURE 5:**
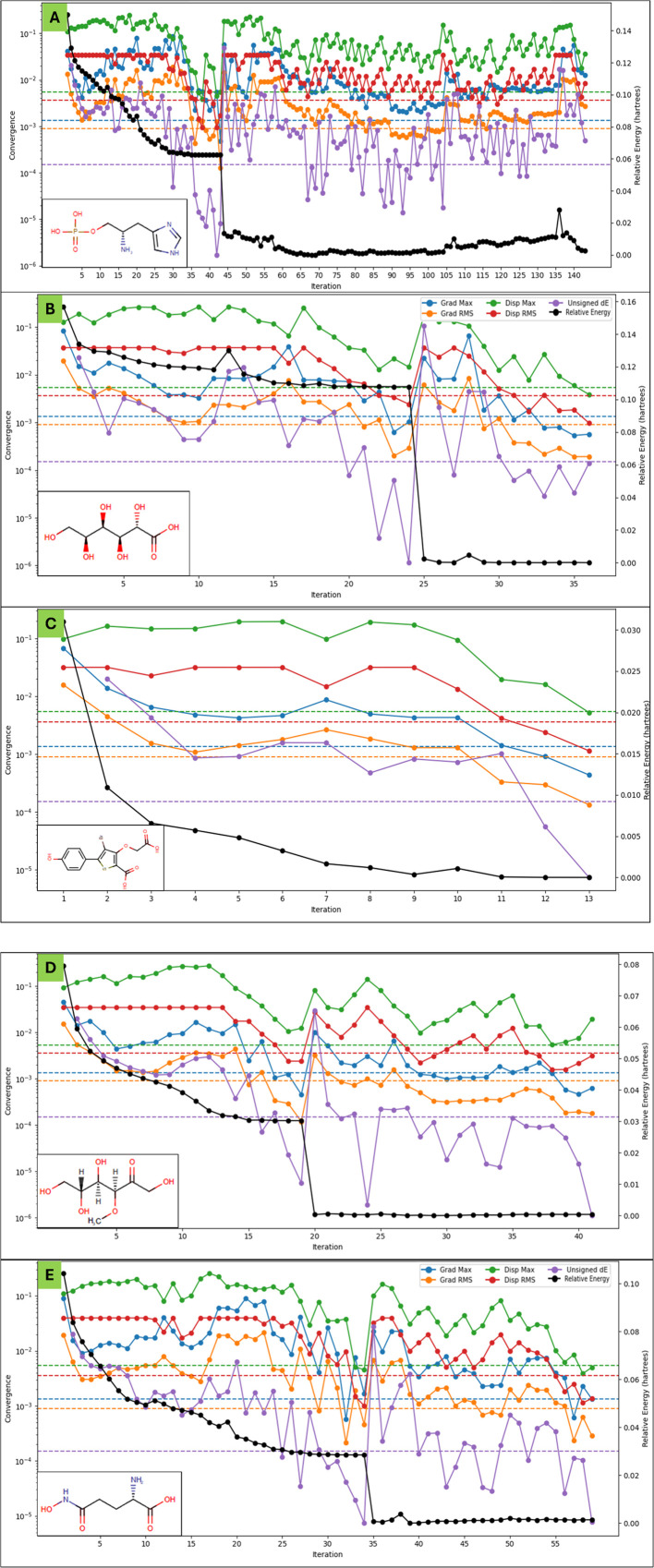
Showing the Density Functional Theory Results for **(A)** L-histidinol phosphate (DB03997), **(B)** L-Gluconic Acid (DB04304), **(C)** 4-bromo-3-(carboxymethoxy)-5-(4-hydroxyphenyl)thiophene-2-carboxylic acid (DB07197), **(D)** 3-O-Methylfructose (DB02438), and **(E)** Glutamine hydroxamate (DB02446) that were identified after molecular docking studies. The legend is shown for different colours with different energies, including the relative energy in black. . .

The maximum ALIE values were highest for DB03997 (382.57 for DB04304 (343.44 kcal/mol). The ALIE mean values were relatively consistent, with DB02446 having the highest average (280.74 kcal/mol) and DB07197 the lowest (246.33 kcal/mol), suggesting that DB07197 may exhibit a slightly lower ionisation threshold. Variance in ALIE was the highest for DB03997, implying greater fluctuation in ionisation potential across the molecule. The average absolute deviation from the mean ALIE followed a similar kcal/mol) and lowest trend, reinforcing the electronic heterogeneity of DB03997. DFT results (Figure 5) highlight stability, reactivity, and electronic properties in the five studied molecules, with DB07197 displaying the highest reactivity and charge separation, while DB03997 exhibited the most pronounced variations in electrostatic and ionisation characteristics.

The pharmacokinetic properties of L-histidinol phosphate, L-Gluconic Acid, 4-Bromo-3-(carboxymethoxy)-5-(4-hydroxyphenyl)thiophene-2-carboxylic acid, 3-O-Methylfructose in linear form, and Glutamine hydroxamate were assessed against standard values to determine their drug-likeness and potential for human absorption (detail in Table 2). The number of rule-of-five violations, which predicts oral bioavailability, was zero for all compounds except L-Gluconic Acid, which had one violation, aligning with the acceptable maximum of four. Similarly, compliance with the rule of three, relevant for fragment-based drug design, was maintained across all molecules except 3-O-Methylfructose in linear form, which did not meet the criteria. The molecular weight of all compounds fell within the standard range of 130.0–725.0 Da, with the highest being 373.174 Da for the thiophene derivative and the lowest at 162.145 Da for Glutamine hydroxamate. The dipole moment values, indicating molecular polarity, were within the standard range of 1.0–12.5, with L-histidinol phosphate (9.725) and the thiophene derivative (9.317) exhibiting a higher polarity, whereas Glutamine hydroxamate (2.261) had the lowest dipole moment, suggesting lower interaction with polar environments. The solvent-accessible surface area (SASA) values, which influence solubility and permeability, were also within the acceptable range of 300.0–1,000.0, with the thiophene derivative having the highest value (522.292) and Glutamine hydroxamate the lowest (358.76). The polar surface area (PSA), crucial for passive diffusion across membranes, ranged from 7.0 to 200.0, with all compounds conforming. However, L-gluconic acid had the highest PSA (152.649), which may indicate lower permeability. The water partition coefficient (QPlogPo/w), reflecting lipophilicity, was below the standard range of −2.0 to 6.5 for L-histidinol phosphate (−2.627), L-Gluconic Acid (−1.879), and Glutamine hydroxamate (−4.578), suggesting poor lipid membrane penetration. In contrast, the thiophene derivative (2.082) was within the acceptable range, indicating a balanced lipophilic profile. Solubility (QPlogS) values ranged between −6.5 and 0.5, where all compounds except the thiophene derivative (−3.506) were within the acceptable limits, confirming their aqueous solubility. Moreover, their intrinsic solubility (CIQPlogS) exhibited similar trends, with the thiophene derivative being the least soluble (−5.114). The prediction of hERG inhibition, which assesses potential cardiotoxicity, showed that none of the compounds had a significant risk, as all QPlogHERG values remained above the critical threshold of −5. Blood-brain barrier permeability (QPlogBB), which predicts CNS activity, indicated that none of the compounds was likely to cross the barrier effectively, with values ranging between −2.358 for L-Gluconic Acid to −1.342 for L-histidinol phosphate, confirming their inactive CNS classification. Intestinal permeability, assessed by QPPCaco, suggested that only 3-O-Methylfructose in linear form (164.605) exhibited moderate absorption potential, while the remaining compounds had very low values (<5), indicating poor permeability. Similarly, hepatic clearance potential (QPPMDCK) was highest for 3-O-Methylfructose in linear form (70.375), suggesting moderate metabolism, whereas the others had low clearance rates. The skin permeability coefficient (QPlogKp) values were below the standard range of −8.0 to −1.0, indicating minimal transdermal absorption potential, with Glutamine hydroxamate being the least permeable (−8.163). Ionisation potential (IP) values related to electronic stability were within the standard range of 7.9–10.5 eV for all compounds except L-Gluconic Acid (11.052), suggesting that it has a higher energy requirement for electron loss. The electron affinity (EA) values, falling between −0.9 and 1.7 eV, were within range for all compounds except the thiophene derivative (1.421), indicating its greater propensity to accept electrons. Metabolic clearance was predicted using the number of metabolism sites (#metab), where 3-O-Methylfructose in linear form had the highest metabolism potential (6), while the others ranged from three to 5, within the expected range of 1–8. Human oral absorption was poor for most compounds, with predicted per cent human oral absorption below 25% for all except the thiophene derivative (45.832%) and 3-O-Methylfructose in linear form (57.964%), which displayed moderate absorption. Plasma protein binding (QPlogKhsa) was negative for all compounds, indicating weak binding affinity, with the thiophene derivative (−0.564) having the least negative value, suggesting slightly higher binding than others. These pharmacokinetic results suggest that none of the studied molecules exhibits high permeability or oral absorption, with 3-O-Methylfructose in a linear form showing the most favourable profile regarding intestinal permeability and metabolism. However, low lipophilicity and poor blood-brain barrier permeability limit their potential for systemic distribution. The thiophene derivative demonstrates moderate absorption and solubility but may require structural modifications to enhance its pharmacokinetic properties.

**TABLE 2:** Showing the Pharmacokinetics of **A)** L-histidinol phosphate (DB03997), **B)** L-Gluconic Acid (DB04304), **C)** 4-bromo-3-(carboxymethoxy)-5-(4-hydroxyphenyl)thiophene-2-carboxylic acid (DB07197), **D)** 3-O-Methylfructose (DB02438), and **E)** Glutamine hydroxamate (DB02446), computed using the QikProp with its standard values.

Descriptors	Standard values	DB03997	DB04304	DB07197	DB02438	DB02446
#stars	0–5	1	2	0	1	3
#amine	0–1	1	0	0	0	1
#amidine	0	0	0	0	0	0
#acid	0–1	2	1	2	0	1
#amide	0–1	0	0	0	0	1
#rotor	0–15	8	10	5	10	6
#rtvFG	0–2	1	0	0	1	1
CNS	−2 (inactive), +2 (active)	−2	−2	−2	−2	−2
mol MW	130.0–725.0	221.152	196.157	373.174	194.184	162.145
dipole	1.0–12.5	9.725	3.257	9.317	3.642	2.261
SASA	300.0–1,000.0	405.621	379.388	522.292	395.846	358.76
FOSA	0.0–750.0	79.443	88.218	28.811	208.199	78.954
FISA	7.0–330.0	225.121	291.17	256.146	187.647	279.806
PISA	0.0–450.0	97.4	0	159.5	0	0
WPSA	0.0–175.0	3.656	0	77.835	0	0
volume	500.0–2000.0	661.691	608.166	891.155	645.048	550.254
donorHB	0.0–6.0	5	5	3	3	5
accptHB	2.0–20.0	8	9.5	5.5	9.5	7.2
dip^2^/V	0.0–0.13	0.142,929	0.0174,382	0.0974,047	0.0205,607	0.0092871
ACxDN^5^/SA	0.0–0.05	0.0441,016	0.0559,919	0.0182,394	0.0415,679	0.044876
glob	0.75–0.95	0.9,053,466	0.9,150,184	0.8574729	0.9,120,817	0.9,051,845
QPpolrz	13.0–70.0	16.12	11.764	27.823	13.24	12.015
QPlogPC16	4.0–18.0	7.901	7.182	10.629	6.357	6.381
QPlogPoct	8.0–35.0	18.62	16.283	18.475	13.419	15.073
QPlogPw	4.0–45.0	16.328	17.087	12.539	13.493	18.113
Type	N/A	small	small	small	small	small
QPlogPo/w	−2.0 – 6.5	−2.627	−1.879	2.082	−1.478	−4.578
QPlogS	−6.5 – 0.5	−0.274	−0.393	−3.506	−0.067	0.942
CIQPlogS	−6.5 – 0.5	−0.265	−0.46	−5.114	−0.252	1.106
QPlogHERG	concern below −5	−0.639	−1.1	−0.984	−2.955	−0.61
QPPCaco	<25 poor, >500 great	1.162	4.348	2.366	164.605	0.742
QPlogBB	−3.0 – 1.2	−1.342	−2.358	−1.869	−1.543	−1.676
QPPMDCK	<25 poor, >500 great	0.624	1.762	3.099	70.375	0.568
QPlogKp	−8.0 to −1.0	−6.62	−5.926	−5.198	−4.018	−8.163
IP(eV)	7.9–10.5	9.04	11.052	9.226	10.692	9.961
EA (eV)	−0.9 – 1.7	−0.605	−0.371	1.421	−0.009	−0.409
#metab	1–8	3	5	4	6	4
QPlogKhsa	−1.5 – 1.5	−1.274	−1.238	−0.564	−1.226	−1.426
HumanOralAbsorption	-	1	2	1	2	1
%HumanOralAbsorption	>80% is high, <25% is poor	12.731	14.41	45.832	57.964	0
SAfluorine	0.0–100.0	0	0	0	0	0
SAamideO	0.0–35.0	0	0	0	0	34.264
PSA	7.0–200.0	122.378	152.649	131.236	112.45	138.28
#NandO	2–15	7	7	6	6	6
RuleOfFive	maximum is 4	0	1	0	0	0
RuleOfThree	maximum is 3	1	1	1	0	1
#ringatoms	-	5	0	11	0	0
#in34	-	0	0	0	0	0
#in56	-	5	0	11	0	0
#noncon	-	0	0	0	0	0
#nonHatm	-	14	13	21	13	11
Jm	-	0.028,238,577	0.094,100,857	0.000988,618	15.99,092,242	0.009,754,937

### 3.5 WaterMap analysis

**FIGURE 6:**
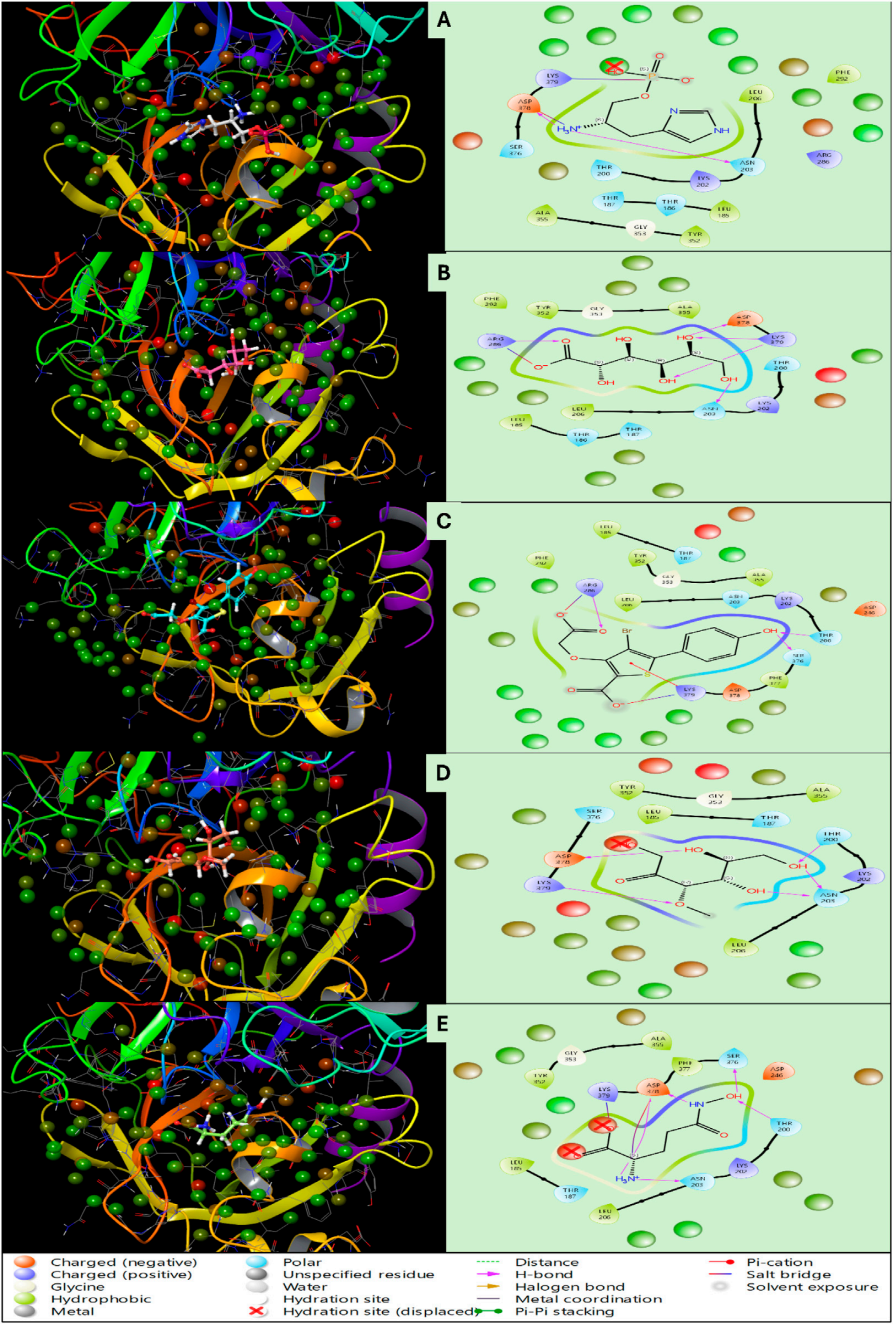
Showing the WaterMap Results in 3D and 2D Ligand Interaction Diagram for Haptoglobin in complex with **(A)** L-histidinol phosphate (DB03997), **(B)** L-Gluconic Acid (DB04304), **(C)** 4-bromo-3-(carboxymethoxy)-5-(4-hydroxyphenyl)thiophene-2-carboxylic acid (DB07197), **(D)** 3-O-Methylfructose (DB02438), and **(E)** Glutamine hydroxamate (DB02446) and legend is shown for various bond and residue types.

Haptoglobin in complex with L-histidinol phosphate (DB03997) shows the ligand-free binding pocket in the hydration landscape. Hydration sites (marked with red Xs) are observed at key regions, primarily near Asp378, Lys379, and Asn203, indicating water molecules occupying these regions without the ligand. These hydration sites suggest areas where solvent molecules form strong hydrogen bonds with charged and polar residues. Thr200, Ser376, and Thr186 also bond hydrogen with surrounding water molecules, contributing to the structural hydration network. Hydrophobic residues such as Leu185, Leu206, and Phe292 exhibit minimal interaction with water molecules, indicating that these regions are less favourable for solvent retention. Haptoglobin in complex with L-Gluconic Acid (DB04304) illustrates the ligand occupying the binding pocket, displacing several hydration sites observed in Figure 6. Key interactions emerge, including hydrogen bonds (purple dashed lines) between the ligand and residues Lys379, Asp378, and Asn203. Additionally, the ligand engages in metal coordination (light blue line) with Lys379, further stabilising the complex. A π-stacking interactions with Phe292 and Tyr352 suggest that the ligand is stabilised through aromatic interactions, reinforcing binding affinity. Hydrophobic residues such as Leu206 and Ala355 provide a non-polar environment that may further contribute to ligand stabilisation. Haptoglobin in complex with 4-bromo-3-(carboxymethoxy)-5-(4-hydroxyphenyl)thiophene-2-carboxylic acid (DB07197) shows a significant displacement of hydration sites is observed due to the ligand’s interaction with key residues. The expelled water molecules (hydration sites marked with red Xs) indicate the ligand’s efficient occupation of these regions, potentially leading to an entropy-driven binding advantage. Salt bridges (solid red lines) are evident between Asp378 and Lys379, contributing to electrostatic stabilisation. Hydrogen bonding interactions with Thr200, Thr186, and Asn203 remain prominent, suggesting a conserved interaction network within the pocket. Additionally, hydrophobic interactions with Tyr352 and Phe292 persist, highlighting the importance of non-polar stabilisation mechanisms. Haptoglobin in complex with 3-O-Methylfructose (DB02438) presents a refined hydration landscape, illustrating how the ligand’s presence leads to a new arrangement of water molecules within the binding pocket. Hydration sites persist near Asp378, Ser376, and Asn203, albeit in altered positions, indicating residual solvent interaction despite ligand binding. The ligand forms additional hydrogen bonds with Ser376 and Thr200, reinforcing its binding stability. A π-cation interaction (red line) involving Arg286 emerges, demonstrating the role of positively charged residues in ligand stabilisation. Metal coordination and hydrogen bonding patterns remain consistent, suggesting that the ligand’s interaction network is well-established at this stage. Haptoglobin in complex with Glutamine hydroxamate (DB02446) shows that the ligand achieves its most stabilised conformation within the binding pocket. Most hydration sites from Figure 6 have been effectively displaced, with the remaining solvent molecules positioned at sites that do not interfere with ligand binding. Salt bridges, hydrogen bonds, and π-π stacking interactions (yellow lines) involving Phe292 and Tyr352 remain intact, indicating substantial aromatic contributions to binding affinity. Hydrophobic interactions persist with Leu185, Leu206, and Ala355, highlighting their role in ligand accommodation. The displacement of hydration sites suggests that the ligand has successfully occupied a previously solvent-filled region, enhancing its thermodynamic favourability. The WaterMap analysis provides valuable insights into the ligand’s interaction landscape, highlighting key molecular forces driving its binding. A combination of hydrogen bonds, salt bridges, metal coordination, and hydrophobic interactions collectively stabilise the ligand within the binding pocket. The displacement of hydration sites suggests that the ligand binding is accompanied by an entropy-driven advantage, reducing the desolvation penalty and enhancing overall affinity. The presence of π-cation and π-π stacking interactions further reinforces ligand stability, particularly in regions involving Phe292 and Tyr352. The overall findings emphasise the importance of hydration site displacement in optimising ligand binding (Figure 6) and suggest that targeting specific hydration pockets could enhance future drug design strategies.

### 3.6 Molecular dynamics simulation analysis

The complete 100ns MD Simulation for all five P-L complexes were analysed using the SID tool for deviation, fluctuations and Intermolecular Interactions analysis. The detailed results are as follows-

#### 3.6.1 Root Mean Square Deviation

**FIGURE 7:**
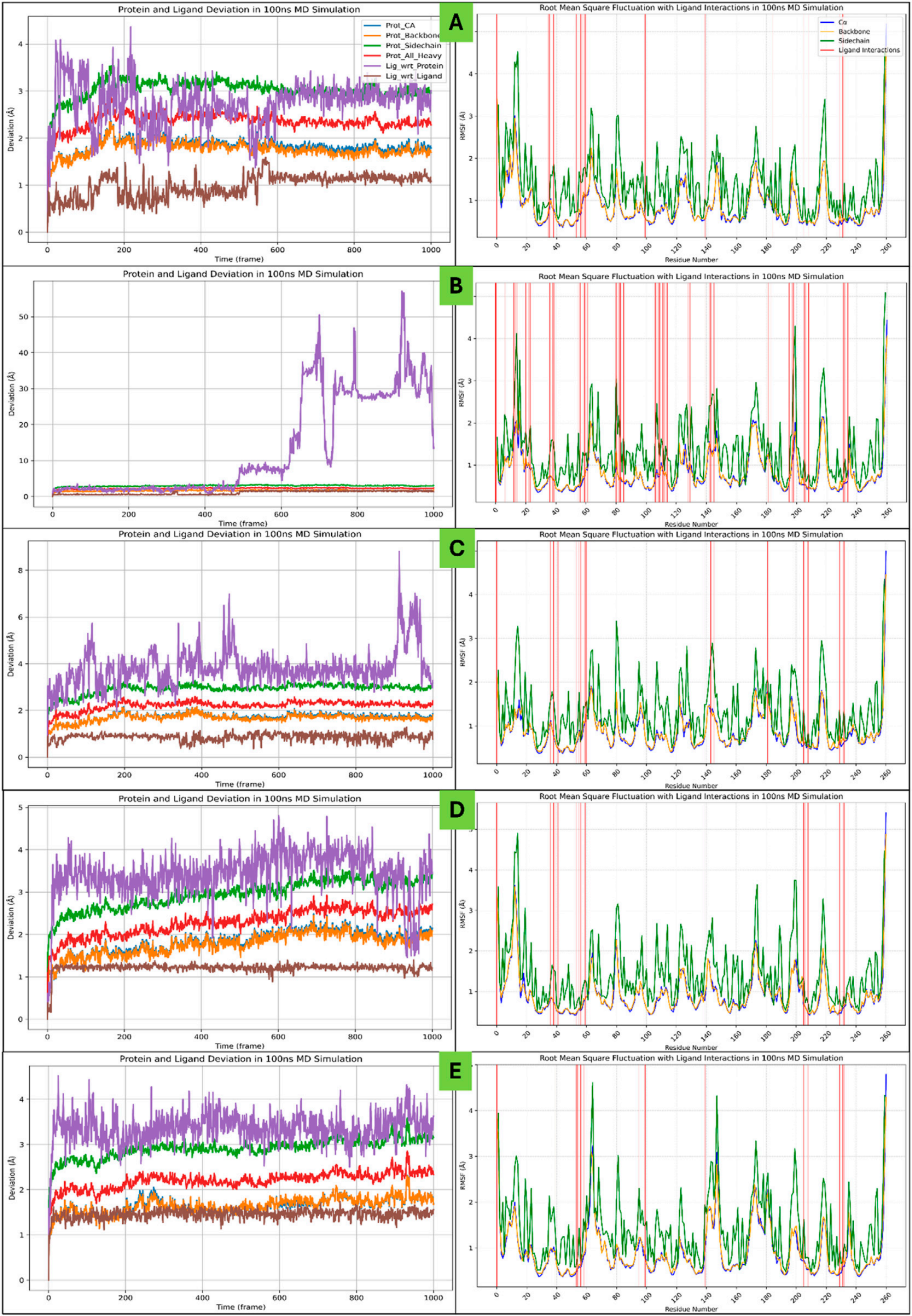
Showing the Molecular Dynamics-based Root Mean Square Deviation (RMSD) and Root Mean Square Fluctuations (RMSF) plots for Haptoglobin in complex with **(A)** L-histidinol phosphate (DB03997), **(B)** L-Gluconic Acid (DB04304), **(C)** 4-bromo-3-(carboxymethoxy)-5-(4-hydroxyphenyl)thiophene-2-carboxylic acid (DB07197), **(D)** 3-O-Methylfructose (DB02438), and **(E)** Glutamine hydroxamate (DB02446). The legend is shown in different colours to differentiate the deviation and fluctuations in the plots.

Root Mean Square Deviation (RMSD) is a critical parameter in molecular dynamics (MD) simulations, providing insights into biomolecular systems’ stability and conformational flexibility. This study analysed the Human haptoglobin protein (PDB ID: 4X0L) in a complex with five different ligands over a 100 ns simulation. The RMSD values of the protein and ligands were assessed at 0.10 ns and 100 ns to determine their structural stability and deviation throughout the simulation. Human haptoglobin in complex with L-Histidinol Phosphate (DB03997), at the beginning of the simulation (0.10 ns), the protein deviated by 0.95 Å, and the ligand showed a deviation of 1.93 Å, indicating an initial phase of adaptation to the simulation environment. The complex exhibited stable performance as the simulation progressed, with fluctuations settling after the equilibration phase. By 100 ns, the protein’s RMSD increased to 1.83 Å, while the ligand reached 2.89 Å. These deviations suggest that while the protein maintained a stable conformation, the ligand experienced moderate movement within the binding site, likely due to flexibility in its interaction network. In the complex of Human Haptoglobin with L-Gluconic Acid (DB04304), the protein initially deviated by 0.92 Å, and the ligand by 1.98 Å at 0.10 ns, reflecting early adjustments in the system. The system displayed consistent stability post-equilibration, with protein RMSD reaching 1.70 Å at 100 ns. However, the ligand showed a significant deviation of 16.18 Å, indicating substantial conformational movement. This high RMSD value suggests that L-Gluconic Acid may have undergone partial dissociation or significant positional rearrangement within the binding site, potentially reflecting weaker interactions with the protein or a high degree of ligand flexibility. In the case of the 4-Bromo-3-(Carboxymethoxy)-5-(4-Hydroxyphenyl) Thiophene-2-Carboxylic Acid with Human Haptoglobin complex, the protein exhibited an RMSD of 1.05 Å, while the ligand deviated by 3.06 Å at 0.10 ns. The simulation indicated a stable interaction profile throughout, with protein RMSD reaching 1.68 Å at 100 ns, demonstrating minimal conformational changes. The ligand RMSD at 100 ns was 2.97 Å, suggesting a relatively well-retained binding pose with slight flexibility. These results indicate stronger retention within the binding site than L-Gluconic Acid but still allow some movement. For the 3-O-Methylfructose in complex with Human Haptoglobin, initial deviations were 0.81 Å for the protein and 0.61 Å for the ligand at 0.10 ns, suggesting minimal fluctuations in the early phase. The system displayed a steady trajectory throughout the simulation, with protein RMSD increasing to 2.02 Å at 100 ns. The ligand showed a deviation of 3.69 Å, indicating a moderate level of mobility. Compared to other complexes, this ligand maintained a relatively stable interaction with the binding site, but its moderate movement suggests some level of dynamic repositioning within the pocket. The Glutamine Hydroxamate with Human Haptoglobin complex demonstrated an initial RMSD of 0.93 Å for the protein and 1.60 Å for the ligand at 0.10 ns, indicating early fluctuations similar to other systems. The complex maintained a stable performance throughout the simulation, with protein RMSD reaching 1.80 Å at 100 ns. The ligand deviated by 3.55 Å, reflecting flexibility comparable to 3-O-Methylfructose. As shown in Figure 7, these results suggest that while the ligand remained within the binding site, it likely exhibited conformational adjustments to optimise interactions.

The RMSD analysis of Human haptoglobin (PDB ID: 4X0L) in a complex with five different ligands provides valuable insights into ligand stability and binding behaviour during a 100 ns molecular dynamics simulation. The protein RMSD remained within the range of 1.68–2.02 Å across all complexes, indicating that the protein structure maintained high stability with only minor fluctuations throughout the simulation. In contrast, the ligand RMSD values exhibited considerable variability, reflecting binding strength and flexibility differences. For instance, L-Histidinol Phosphate, 4-Bromo-3-(Carboxymethoxy)-5-(4-Hydroxyphenyl) Thiophene-2-Carboxylic Acid, and Glutamine Hydroxamate demonstrated moderate ligand RMSD values between 2.89 and 3.69 Å, suggesting that these ligands retained interactions with the protein while undergoing some movement. On the other hand, L-Gluconic Acid displayed an unusually high ligand RMSD of 16.18 Å, indicating substantial movement that may indicate weak binding affinity or partial dissociation from the binding site. Meanwhile, 3-O-Methylfructose showed moderate ligand movement (3.69 Å), suggesting a balance between stability and flexibility within the protein’s binding pocket. These findings highlight that L-Gluconic Acid exhibited the least stable binding and may require further optimisation to improve its interaction with the protein. In contrast, ligands with RMSD values below 4 Å, such as L-Histidinol Phosphate and Glutamine Hydroxamate, demonstrated stronger retention within the binding site, making them promising candidates for further drug development studies.

#### 3.6.2 Root Mean Square Fluctuations

The Root Mean Square Fluctuation (RMSF) analysis of Human Haptoglobin (PDB ID: 4X0L) in complex with different ligands was conducted to assess the flexibility of individual amino acid residues throughout the MD simulation. Higher RMSF values indicate protein regions that exhibit greater fluctuations, which could correspond to loop regions, flexible binding sites, or unstable interactions. The analysis also identified residues involved in stable interactions with the ligand, contributing to the overall stability of the complex. In the Human Haptoglobin - L-Histidinol Phosphate (DB03997) complex, several residues exhibited significant fluctuations beyond 2 Å, including VAL148, ALA156, VAL159, GLN160, GLU210, ASN211, and ASN406. These residues are likely part of the protein’s loop or solvent-exposed regions that exhibit natural flexibility during the simulation. Despite these fluctuations, multiple residues contributed to stabilising interactions with the ligand, including SER181, HIS182, HIS183, LEU185, THR186, THR200, LYS202, ASN203, LEU204, PHE205, LEU206, ASN207, ASP246, ARG286, GLN331, TYR352, ALA355, SER376, ASP378, and LYS379. Multiple hydrogen bond donors and acceptors within these stabilising residues suggest a well-retained ligand in the binding site, compensating for the flexibility observed in certain protein regions. For the Human Haptoglobin - L-Gluconic Acid (DB04304) complex, residues GLN160, ARG161, LEU163, VAL318, GLU320, TYR346, LEU364, GLU365, and ASN406 fluctuated beyond 2 Å, indicating considerable structural flexibility in these regions. The presence of fluctuations in residues involved in ligand binding, such as GLN160 and ARG161, suggests that the ligand-induced conformational changes contributed to the observed flexibility. Despite this, the ligand formed stable interactions with a large number of residues, including LYS153, VAL159, GLN160, ARG161, LEU167, ALA169, LYS170, HIS183, ASN184, LEU185, THR186, LYS202, ASN203, PHE205, LEU206, ASN207, HIS208, LYS227, LYS228, GLN229, LEU230, VAL231, GLU232, LYS253, GLN254, LYS255, VAL256, SER257, VAL258, ASN259, GLU260, ARG261, VAL275, GLY276, ARG286, ASN289, PHE290, PHE292, VAL328, VAL330, GLY342, SER344, LYS345, TYR352, GLY353, ALA355, ASP378, LYS379, and CYS381. The extensive interaction network suggests that while certain protein regions were flexible, the ligand maintained strong contact with critical binding residues, contributing to the complex’s overall stability. In the case of Human Haptoglobin - 4-Bromo-3-(Carboxymethoxy)-5-(4-Hydroxyphenyl) Thiophene-2-Carboxylic Acid (DB07197), ASN406 was the only residue fluctuating beyond 2 Å, indicating that the majority of the protein structure remained highly stable. The ligand maintained interactions with several residues, including HIS183, LEU185, GLY188, THR200, LYS202, ASN203, PHE205, LEU206, ASN207, ARG286, PHE290, PHE292, VAL328, TYR352, ALA355, SER376, ASP378, and LYS379. The fact that only a single residue exhibited substantial fluctuation suggests that this ligand was particularly effective in maintaining the conformational integrity of the protein, potentially making it a strong candidate for further optimisation in drug design. For the Human Haptoglobin - 3-O-Methylfructose (DB02438) complex, fluctuations beyond 2 Å were observed in residues VAL148, PRO158, VAL159, GLN160, ARG161, LYS227, GLU320, LYS321, and ASN406. These fluctuations indicate flexibility primarily in loop and surface regions. However, stabilising interactions were formed by residues HIS183, LEU185, THR186, GLY188, THR200, LYS202, ASN203, LEU206, ARG286, TYR352, GLY353, ALA355, SER376, ASP378, and LYS379. The presence of both flexible and stable residues suggests that while certain areas of the protein were structurally dynamic, the ligand remained tightly associated with key binding site residues. Finally, in the Human Haptoglobin –

Glutamine Hydroxamate (DB02446) complex, residues VAL148, GLU210, ASN211, ALA212, ASP294, PRO319, GLU320, VAL328, and ASN406 exhibited significant fluctuations beyond 2 Å, indicating localised structural flexibility. Despite these fluctuations, the ligand formed stable interactions with THR186, THR200, ALA201, LYS202, ASN203, PHE205, TYR242, ASP246, ARG286, TYR352, ALA355, SER376, ASP378, and LYS379. The presence of key stabilising residues within the binding pocket suggests that while specific protein regions were highly flexible, the ligand-binding domain remained relatively stable, ensuring a strong ligand-protein interaction, as shown in Figure 7. The RMSF highlights key differences in the structural dynamics of Human Haptoglobin (PDB ID: 4X0L) complexes with different ligands. While certain protein regions exhibited notable flexibility, the presence of well-retained ligand-protein interactions in most cases suggests that ligand stability within the binding pocket was maintained. Notably, the L-Gluconic Acid complex exhibited the highest number of fluctuating residues, suggesting weaker ligand retention or increased conformational changes upon binding. In contrast, the 4-Bromo-3-(Carboxymethoxy)-5-(4-Hydroxyphenyl) Thiophene-2-Carboxylic Acid complex displayed minimal fluctuation, suggesting a more rigid and stable interaction with the protein. These findings provide valuable insights into these ligand-protein complexes’ binding stability and flexibility, which can be useful for designing and optimising future drug candidates targeting Human Haptoglobin.

#### 3.6.3 Simulation interaction diagram

**FIGURE 8:**
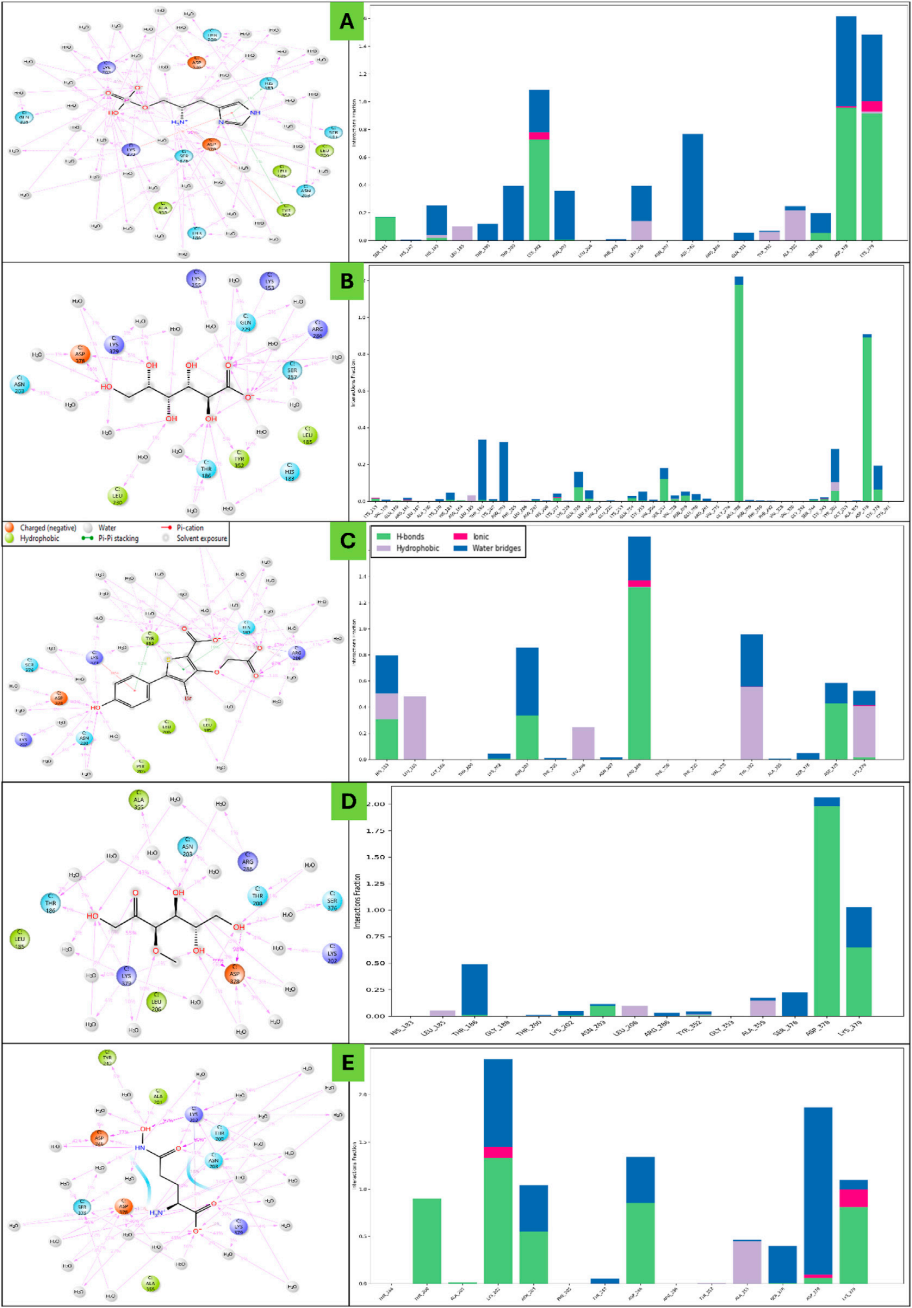
Showing the Molecular Dynamics-based Simulation Interaction Diagram (SID) and its histogram representations for haptoglobin in complex with **(A)** L-histidinol phosphate (DB03997), **(B)** L-Gluconic Acid (DB04304), **(C)** 4-bromo-3-(carboxymethoxy)-5-(4-hydroxyphenyl)thiophene-2-carboxylic acid (DB07197), **(D)** 3-O-Methylfructose (DB02438), and **(E)** Glutamine hydroxamate (DB02446). The legend is in different colours to differentiate the plots’ bonds and residue types.

The Simulation Interaction Diagram (SID) analysis of Human Haptoglobin (PDB ID: 4X0L) in complex with different ligands provides insights into the stability and nature of the interactions formed during the molecular dynamics simulation. The study reveals the presence of key hydrogen bonds, water-mediated interactions, salt bridges, and π-stacking interactions, which contribute to stabilising the ligand-protein complexes. In the Human Haptoglobin - L-Histidinol Phosphate (DB03997) complex, multiple hydrogen bonds were observed, primarily involving ASP246, ASN203, and THR200 residues, which formed interactions with water molecules through the N + H3 atom of the ligand. Additionally, residues such as HIS183, LEU206, ASN203, LYS202, ASP378, and THR186 contributed to hydrogen bonding with water molecules, enhancing the stability of the complex. The SER181 residue established two hydrogen bonds via its N atoms, while LYS379 and LYS202 residues also formed interactions with water molecules, along with ASP378, which interacted via two O atoms. A salt bridge interaction was observed between ASP378 and the N + H3 atom of the ligand, further stabilising the complex. These interactions highlight the significant role of both direct hydrogen bonds and water-mediated interactions in maintaining ligand binding, reinforcing the stability of the protein-ligand complex throughout the simulation. For the Human Haptoglobin - L-Gluconic Acid (DB04304) complex, hydrogen bond interactions were observed between ASP378, SER257, and GLN229 residues, while LYS379, ASN203, LEU230, HIS183, and THR186 residues formed additional water-mediated interactions with five OH atoms of the ligand. Furthermore, TYR352, GLN229, LYS153, and ARG286 residues, along with LYS255, engaged in hydrogen bonding interactions via two O atoms of the ligand. The involvement of multiple residues in water-mediated interactions suggests a highly hydrated and dynamic binding environment where the ligand is stabilised through an extensive hydrogen bond network. These interactions are critical in maintaining ligand orientation within the binding site, compensating for any fluctuations observed in the molecular dynamics simulation. In the Human Haptoglobin - 4-Bromo-3-(Carboxymethoxy)-5-(4-Hydroxyphenyl) Thiophene-2-Carboxylic Acid (DB07197) complex, hydrogen bond interactions were detected between ASN203 and ASP378 residues, while LYS202 and LYS379 residues engaged in water-mediated interactions via the OH atom. ARG286 and TYR352 residues also formed water-mediated hydrogen bonds, while HIS183 interacted with five O atoms, suggesting a strong electrostatic interaction profile within the binding site. Notably, three π-π stacking interactions were observed, involving HIS183 and TYR352 residues with two benzene rings of the ligand. Additionally, a π-cation interaction was formed between LYS379 and the benzene ring of the ligand, contributing to further stabilisation. A salt bridge interaction was also identified between ARG286 and the O atom of the ligand, reinforcing ligand retention within the binding site. A π-stacking and salt bridge interactions suggest a strong, multi-faceted binding mechanism, where electrostatic and hydrophobic interactions play a significant role in ligand stabilisation. For the Human Haptoglobin - 3-O-Methylfructose (DB02438) complex, hydrogen bonding interactions were established between ASN203, THR186, LYS379, and ASP378 residues, while SER376 and LYS202 residues engaged in interactions with the ligand through four OH atoms. Additionally, two O atoms of the ligand interacted with the LYS379 residue, suggesting a significant contribution of water-mediated interactions to ligand stabilisation. Compared to other complexes, the relatively simpler interaction profile indicates that hydrogen bonding is dominant in maintaining ligand binding. The absence of π-stacking or salt bridge interactions suggests that this ligand relies primarily on polar and electrostatic interactions for stability within the binding pocket. In the Human Haptoglobin - Glutamine Hydroxamate (DB02446) complex, multiple hydrogen bond interactions were observed, involving ASP378, ASN203, and LYS202 residues, which interacted with water molecules through the N + H3 atom. Additional interactions were formed by ASP246 and SER376 residues via the NH atom, while the OH atom of the ligand engaged ASP246, LYS202, and TYR242 residues through water-mediated interactions. Moreover, three oxygen atoms of the ligand participated in hydrogen bonding interactions with LYS379, THR200, LYS202, ASN203, ASP378, and SER376 residues, highlighting a densely connected hydrogen bond network. Notably, three salt bridges were formed, involving ASP378 with the N + H3 atom and LYS202 and LYS379 with the O atom of the ligand. Multiple salt bridges suggest a strong electrostatic interaction profile, which, combined with the extensive hydrogen bonding network, reinforces ligand stability and retention within the binding site. The Simulation Interaction Diagram (SID) analysis highlights the diverse interaction profiles of different ligand-protein complexes. L-histidinol phosphate and L-gluconic acid demonstrated extensive water-mediated hydrogen bonding, contributing to ligand stability. The 4-Bromo-3-(Carboxymethoxy)-5-(4-Hydroxyphenyl) Thiophene-2-Carboxylic Acid complex showed a unique combination of hydrogen bonding, π-stacking, and salt bridge interactions, indicating a highly stable binding conformation. 3-O-Methylfructose primarily relied on hydrogen bonding interactions, while Glutamine Hydroxamate exhibited an extensive hydrogen bond network alongside three salt bridge interactions, further reinforcing ligand stability. These findings emphasise the importance of hydrogen bonding, salt bridges, and π-stacking interactions in determining ligand stability and affinity, providing valuable insights into potential structure-based drug design strategies targeting Human Haptoglobin. Further, Figure 8 shows the SID and histogram representation for the same to understand the interactions better.

### 3.7 Analysis of binding free energy

**FIGURE 9:**
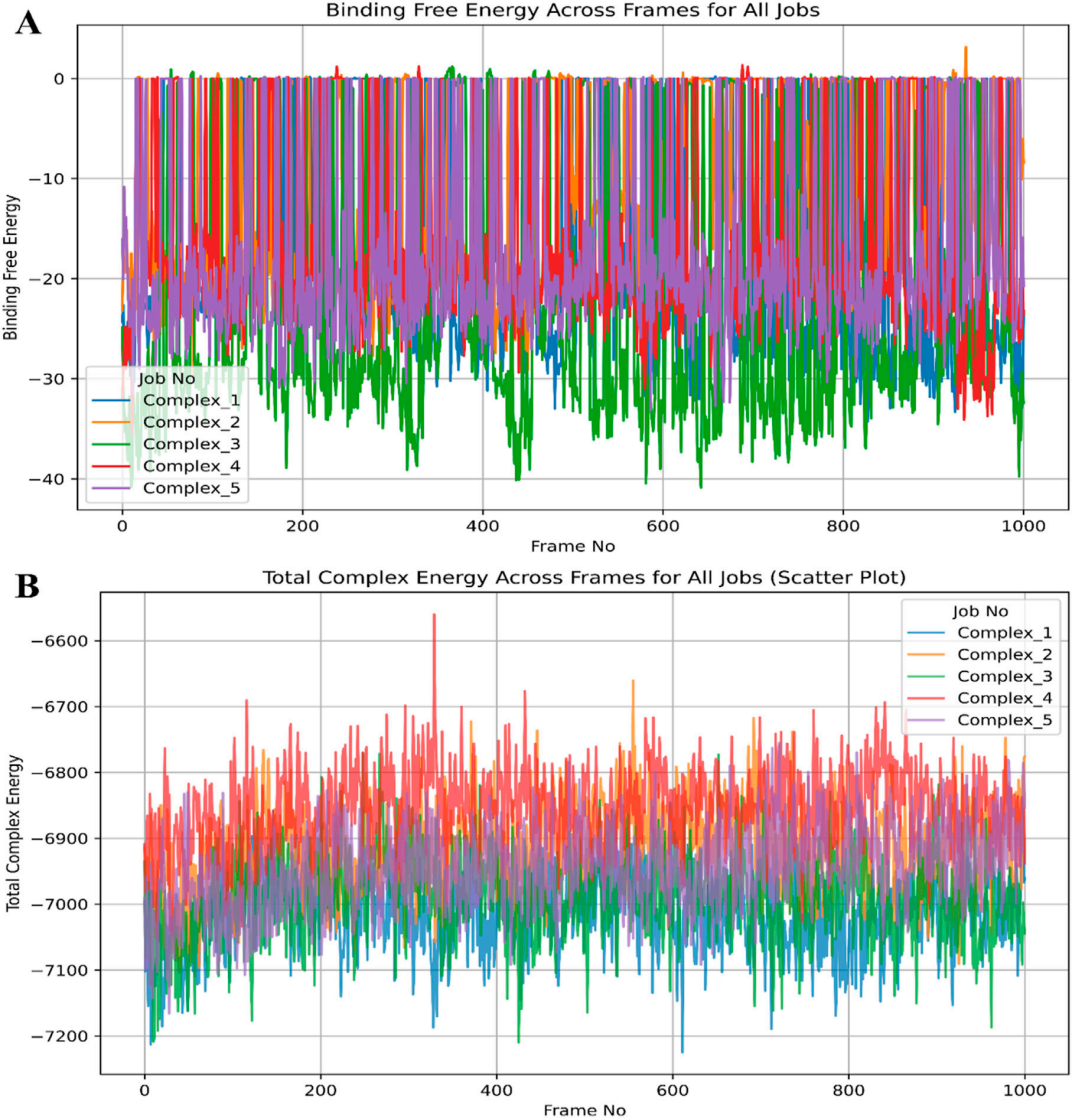
Showing the molecular dynamics-based molecular mechanics with generalised born and surface area solvation (MM\GBSA) computations plots for **(A)** binding free energy and **(B)** total complex energy for haptoglobin in complex with L-histidinol phosphate (DB03997), L-Gluconic Acid (DB04304), 4-bromo-3-(carboxymethoxy)-5-(4-hydroxyphenyl)thiophene-2-carboxylic acid (DB07197), 3-O-Methylfructose (DB02438), and Glutamine hydroxamate (DB02446). The legend is shown in different colours to differentiate energies.

The MM\GBSA binding free energy calculations provide insights into the binding affinity and stability of various ligand complexes with human haptoglobin (PDB ID: 4X0L) based on MD simulation trajectories. The calculations were performed using the mmgbsa. py script, and the results were analysed in terms of ΔG (binding free energy), its standard deviation, and range across 1,001 frames. Both dG (standard binding free energy) and dG (NS) (non-standard binding free energy) values were evaluated for each ligand-protein complex. The binding free energy (dG) for L-Histidinol Phosphate in complex with haptoglobin was calculated as −17.4661 kcal/mol on average, indicating a moderately stable binding interaction. The standard deviation of 11.54 kcal/mol and the range from −34.2750 to 0.2085 kcal/mol suggest significant fluctuations in binding affinity during the MD simulation, with instances of both strong and weak binding interactions. The non-standard dG (dG NS) exhibited a slightly more negative average of −19.0667 kcal/mol, with a higher standard deviation of 12.54 kcal/mol, extending from −35.8764 to 0.0320 kcal/mol. These variations indicate some degree of structural flexibility and potential ligand reorientation within the binding site. Despite these fluctuations, the negative binding free energy values confirm that L-Histidinol Phosphate maintains a favourable interaction with haptoglobin, likely facilitated by hydrogen bonding and salt bridge formation observed in the interaction analysis. The L-Gluconic Acid in a complex with haptoglobin exhibited a weaker binding affinity, with an average binding free energy of −11.0846 kcal/mol and a standard deviation of 9.07 kcal/mol. The dG range spanned from −29.5777 to 3.1576 kcal/mol, indicating transient favourable and weak binding interactions during the MD simulation. The non-standard binding free energy (dG NS) was calculated as −12.0982 kcal/mol, with a slightly higher standard deviation of 9.83 kcal/mol and a broader range of −34.1693 to 0.7836 kcal/mol. Positive values in the dG range suggest that in some simulation frames, L-Gluconic Acid may exhibit partial dissociation from the binding site, possibly due to a less optimal interaction network. This aligns with the interaction analysis, where water-mediated hydrogen bonds dominated ligand stabilisation rather than direct strong polar or hydrophobic contacts. Among the analysed complexes, the 4-Bromo-3-(Carboxymethoxy)-5-(4-Hydroxyphenyl) Thiophene-2-Carboxylic Acid in complex with haptoglobin displayed the strongest binding affinity, with an average dG of −21.3262 kcal/mol and a standard deviation of 14.44 kcal/mol. The range extended from −40.8947 to 1.2106 kcal/mol, suggesting instances of very strong binding, though some fluctuations occurred. The non-standard binding free energy (dG NS) was even more negative at −22.6810 kcal/mol, with a higher standard deviation of 15.34 kcal/mol and a binding range of −43.7957 to 1.2326 kcal/mol. These results indicate that this ligand exhibited the most favourable interactions with haptoglobin, consistent with its strong hydrogen bonding, salt bridge formation, and π-π stacking interactions. A π-stacking interaction with HIS183 and TYR352 and a π-cation interaction with LYS379 likely contributed to its superior binding stability. The 3-O-Methylfructose in complex with haptoglobin exhibited moderate binding stability, with an average dG of −15.9200 kcal/mol and a standard deviation of 9.75 kcal/mol. The binding energy range spanned from −35.4498 to 1.3493 kcal/mol, suggesting notable fluctuations in ligand stability within the binding site. The non-standard dG (dG NS) was calculated as −18.8021 kcal/mol, with a higher standard deviation of 11.41 kcal/mol and a range from −43.4431 to 0.0084 kcal/mol. The observed fluctuations suggest some degree of flexibility in ligand positioning, with hydrogen bonding as the primary interaction mode. The absence of π-stacking and salt bridges may explain the slightly lower binding affinity than DB07197, but it is still within a favourable range for stable binding. The Glutamine Hydroxamate in complex with haptoglobin showed a binding free energy of −16.6191 kcal/mol, with a standard deviation of 9.96 kcal/mol. The range extended from −33.1633 to 0.2528 kcal/mol, indicating moments of both strong and weak binding interactions. The non-standard dG (dG NS) was calculated as −19.0530 kcal/mol, with a higher standard deviation of 11.27 kcal/mol and a range from −37.3372 to 0.0120 kcal/mol. Three salt bridges (ASP378-NH3, LYS202-O, and LYS379-O) and an extensive hydrogen bonding network contributed to its favourable binding affinity. The relatively high standard deviation suggests dynamic interactions, likely due to ligand flexibility within the binding pocket. The MM/GBSA binding free energy results reveal distinct interaction profiles and stability trends across ligand-haptoglobin complexes. Among the studied ligands, 4-Bromo-3-(Carboxymethoxy)-5-(4-Hydroxyphenyl) Thiophene-2-Carboxylic Acid (DB07197) exhibited the most stable binding (−21.3262 kcal/mol dG, −22.6810 kcal/mol dG NS), likely due to multiple interaction types (hydrogen bonding, salt bridges, π-stacking, and π-cation interactions). In contrast, L-Gluconic Acid (DB04304) exhibited the weakest binding affinity (−11.0846 kcal/mol dG), characterised primarily by water-mediated hydrogen bonds rather than direct electrostatic or hydrophobic interactions. The L-Histidinol Phosphate (DB03997), 3-O-Methylfructose (DB02438), and Glutamine Hydroxamate (DB02446) complexes exhibited moderate binding affinities, with Glutamine Hydroxamate showing strong salt bridge interactions contributing to stability (Figure 9). The standard deviation and binding energy ranges across all complexes indicate that ligand binding fluctuates dynamically during MD simulations, with some ligands displaying transient weaker binding states before re-establishing stronger interactions. These findings provide crucial insights into ligand stability, binding mechanisms, and potential drug design optimisations for haptoglobin-targeted therapeutic strategies.

## 4 Discussion

Haptoglobin (Hp) is a vital glycoprotein primarily involved in haemoglobin binding and clearance, thereby preventing oxidative stress and inflammation. Besides its crucial physiological role, Hp has been implicated in various pathophysiological conditions, including cancer, infections, and autoimmune disorders. Given its multifaceted biological significance, the modulation of Hp activity through small-molecule ligands has gained substantial attention in therapeutic drug discovery. Identifying ligands that exhibit strong and specific binding to Hp can pave the way for novel pharmacological interventions to modulate its function in disease contexts. This study employed an integrative computational framework to characterise the binding affinities, interaction fingerprints, electronic properties, pharmacokinetics, and dynamic behaviour of five potential ligands targeting Hp. The clinical relevance of Hp extends beyond its haemoglobin-binding activity, as it plays a role in immune modulation, inflammation, and metabolic disorders. Aberrant expression and function of Hp have been associated with various diseases, including cardiovascular complications, infectious diseases, and cancer. The ability to pharmacologically regulate Hp function via small-molecule interactions presents an opportunity to develop targeted therapies. Ligands that can interact with Hp at a molecular level might help modulate its activity, potentially leading to therapeutic applications in haemolytic conditions, sepsis, and inflammatory diseases. In cancer, the upregulation of haptoglobin has been associated with poor prognosis, particularly in haematological malignancies such as lymphoma and leukaemia. The elevation of haptoglobin levels in these cancers may be linked to its ability to promote an inflammatory microenvironment that aids tumour progression. Haptoglobin’s capacity to suppress immune responses and its potential role in promoting tumour angiogenesis are key factors in its involvement in malignancy. Furthermore, the modulation of haptoglobin could present an opportunity for targeted therapeutic strategies to reverse the tumour-promoting effects of haptoglobin while maintaining its essential functions in preventing oxidative damage and haemoglobin clearance. Haptoglobin-targeted therapies could offer a dual benefit in malignancies: one in reducing the oxidative stress associated with haemolysis in cancer patients and the other in mitigating the immunosuppressive effects of haptoglobin that facilitate tumour growth. Ongoing research into haptoglobin’s role in cancer provides an exciting opportunity to develop therapeutic interventions that specifically modulate haptoglobin’s effects in the context of cancer, improving patient outcomes. Given this significance, our study aimed to computationally evaluate the binding potential of various small molecules with Hp, offering an in-depth understanding of their interaction dynamics and stability. Hp (PDB ID: 4X0L) was retrieved from the Protein Data Bank and prepared systematically to ensure the accuracy of computational docking and molecular simulations. This included energy minimisation, removal of crystallographic water molecules, and addition of missing hydrogen atoms to optimise the structure for docking and simulation. The protein structure was then optimised for molecular mechanics to maintain its native conformation during molecular docking and MD simulations.

Molecular docking studies were performed that have helped to sort the best five binding affinities of small molecules—L-Histidinol Phosphate (DB03997), L-Gluconic Acid (DB04304), 4-Bromo-3-(Carboxymethoxy)-5-(4-Hydroxyphenyl) Thiophene-2-Carboxylic Acid (DB07197), 3-O-Methylfructose (DB02438), and Glutamine Hydroxamate (DB02446)—with Hp. The docking results revealed favourable binding affinities, with docking scores indicating high stability of the ligand-protein complexes. Interaction analysis identified key hydrogen bonding, salt bridge formations, and π-stacking interactions that contributed to the ligand stability within the active site of Hp. Among the analysed compounds, DB07197 exhibited the strongest binding affinity, attributed to multiple stabilising interactions, including hydrogen bonding with ASN203 and ASP378, π-π stacking with HIS183 and TYR352, and salt bridge formation with ARG286. These interactions suggested that DB07197 forms an extensive interaction network, contributing to enhanced stability. In contrast, L-Gluconic Acid (DB04304) showed the weakest binding affinity, primarily stabilised by water-mediated hydrogen bonds rather than direct electrostatic or hydrophobic interactions. The interaction fingerprints for the remaining ligands suggested moderate binding affinity, with hydrogen bonding playing a key role in stabilising the complexes. To gain deeper insights into the electronic properties of the ligands and their potential reactivity, Density Functional Theory (DFT) calculations were performed. The HOMO-LUMO energy gap analysis indicated the stability and reactivity of each ligand, providing insights into their electronic transitions and binding potential. Ligands with lower HOMO-LUMO gaps were identified as chemically more reactive, suggesting more substantial interaction potential with Hp. The molecular electrostatic potential (MEP) maps further revealed the charge distribution, helping to understand the nucleophilic and electrophilic regions of the ligands, which influence their binding efficacy. DB07197 displayed a favourable electronic distribution with a balanced charge density around its active functional groups, contributing to its strong interaction with Hp. ADMET (Absorption, Distribution, Metabolism, Excretion, and Toxicity) analysis was performed to assess the drug-likeness and pharmacokinetic potential of the selected ligands. The pharmacokinetic predictions suggested that most ligands exhibited high gastrointestinal (GI) absorption, moderate blood-brain barrier (BBB) permeability, and favourable metabolic stability. Notably, DB07197 demonstrated a high GI absorption rate, indicating good oral bioavailability, while L-Gluconic Acid exhibited poor BBB permeability, limiting its application in central nervous system (CNS)-related conditions. None of the ligands showed significant toxicity concerns, supporting their potential as viable drug candidates.

WaterMap analysis was conducted to assess the contribution of solvent dynamics to ligand binding. The displacement of high-energy water molecules in the Hp binding site was analysed to understand solvent-mediated effects on ligand stability. The results indicated that DB07197 efficiently displaced unfavourable water molecules, enhancing binding affinity through enthalpic contributions. In contrast, L-Gluconic Acid retained many water molecules, potentially contributing to its weaker binding affinity. The WaterMap results provided valuable insights into the solvation effects that influence ligand stability, further reinforcing the docking and MD simulation findings. To evaluate the dynamic behaviour and stability of the ligand-protein complexes over time, MD simulations were conducted for 100 ns. The root-mean-square deviation (RMSD), root-mean-square fluctuation (RMSF), radius of gyration (Rg), and solvent-accessible surface area (SASA) were analysed to assess the structural integrity and flexibility of the complexes. The DB07197 complex displayed the lowest RMSD fluctuations, indicating a highly stable binding conformation. Conversely, L-Gluconic Acid showed higher RMSD values, suggesting greater conformational flexibility and reduced stability. The RMSF analysis revealed minimal fluctuations in key binding site residues, confirming the robustness of ligand interactions. MM/GBSA calculations were performed using mmgbsa. py on the MD trajectories to quantify the binding affinity of each ligand. The binding free energy (ΔG) values provided insights into the energetic favourability of each complex. DB07197 exhibited the most favourable ΔG value (−21.3262 kcal/mol), indicating the strongest binding affinity. The high stability of this complex was attributed to strong electrostatic interactions, hydrogen bonding, and salt bridge formation. On the other hand, L-Gluconic Acid exhibited the weakest binding affinity (−11.0846 kcal/mol), reflecting its weaker interaction profile and higher solvation effects. The other ligands demonstrated moderate binding affinities, consistent with their interaction networks observed in docking and MD simulations. This study presents a comprehensive computational analysis of five potential ligands targeting haptoglobin, integrating molecular docking, MD simulations, DFT analysis, pharmacokinetics, WaterMap analysis, and MM/GBSA calculations. DB07197 emerged as the most promising candidate among the studied ligands, exhibiting strong binding affinity, favourable pharmacokinetic properties, high electronic stability, and minimal structural fluctuations during MD simulations. These findings provide a strong foundation for further *in vitro* and *in vivo* validation studies to explore the therapeutic potential of these ligands in modulating haptoglobin function for disease intervention. Future studies may focus on lead optimisation, structural modifications, and experimental validation to develop potent haptoglobin-targeted therapeutics.

## 5 Conclusion

This study comprehensively investigated the binding potential and stability of five drug-like molecules with human haptoglobin (PDB ID: 4X0L) using a variety of computational techniques, including molecular docking, interaction fingerprinting, DFT, pharmacokinetic profiling, WaterMap analysis, MD simulations, and MM/GBSA free energy calculations. Molecular docking revealed strong binding affinities ranging from −7.96 to −5.58 kcal/mol, with extensive interactions at key residues. Interaction fingerprinting and DFT analysis confirmed the presence of stable hydrogen bonds, salt bridges, and π–π stacking interactions, contributing to ligand affinity and stability. The pharmacokinetic evaluation indicated that all ligands met drug-likeness criteria with favourable ADME profiles. WaterMap analysis identified critical hydration sites that influence ligand binding. MD simulation results confirmed the stability of the protein-ligand complexes, with RMSD values stabilising between 1.68 Å and 2.02 Å for the protein and 2.89 Å to 16.18 Å for the ligands. RMSF analysis highlighted key fluctuating residues, while simulation interaction diagrams validated strong hydrogen bonding networks. MM/GBSA calculations indicated that DB07197 had the most favourable free energy (−21.32 kcal/mol), followed by DB03997 (−17.46 kcal/mol). These findings underscore DB07197 and DB03997 as promising candidates for further exploration as haptoglobin inhibitors. Given the promising computational results, these compounds warrant *in vitro* and *in vivo* validation to assess their efficacy and safety. Furthermore, future research should focus on exploring the effects of haptoglobin polymorphisms on these compounds’ binding affinity and therapeutic potential and investigating their broader implications in disease models. This study’s integration of advanced computational methods provides a robust framework for rational drug discovery and offers a pathway for developing targeted therapies that modulate haptoglobin activity. Identifying and validating effective haptoglobin modulators could open new therapeutic avenues for treating haemolysis, inflammation, and immune modulation conditions.

## Data Availability

The original contributions presented in the study are included in the article/Supplementary Material, further inquiries can be directed to the corresponding authors.

## References

[B1] Ahmad S. Bano N. Khanna K. Gupta D. Raza K. (2024a). Reporting multitargeted potency of Tiaprofenic acid against lung cancer: molecular fingerprinting, MD simulation, and MTT-based cell viability assay studies. Int. J. Biol. Macromol. 276, 133872. 10.1016/j.ijbiomac.2024.133872 39019378

[B2] Ahmad S. Bano N. Qazi S. Yadav M. K. Ahmad N. Raza K. (2022a). Multitargeted molecular dynamic understanding of butoxypheser against SARS-CoV-2: an *in silico* study. Nat. Product. Commun. 17, 1934578X221115499. 10.1177/1934578x221115499

[B3] Ahmad S. Bano N. Raza K. (2025a). Evaluating the polypharmacological potency of FEDPN from ChEMBL BioAssays against lung cancer EGFR, ALK, TrkA and KRAS proteins. Int. J. Biol. Macromol. 306, 141703. 10.1016/j.ijbiomac.2025.141703 40043981

[B4] Ahmad S. Bano N. Raza K. (2025b). RCSB Protein Data Bank: revolutionising drug discovery and design for over five decades. Med. Data Min. 8, 8–11. 10.53388/mdm202508008

[B5] Ahmad S. Dahiya V. Vibhuti A. Pandey R. P. Tripathi M. K. Yadav M. K. (2023a). “Therapeutic protein-based vaccines,” in Protein-based therapeutics (Singapore Singapore: Springer Nature), 355–384.

[B6] Ahmad S. Kishan A. Chitkara P. Asiri S. A. Eswaran M. Mehta S. (2023b). “Natural product-based drug designing for treatment of human parasitic diseases,” in Natural product based drug discovery against human parasites: opportunities and challenges (Springer), 37–59.

[B7] Ahmad S. Pasha Km M. Raza K. Rafeeq M. M. Habib A. H. Eswaran M. (2023c). Reporting dinaciclib and theodrenaline as a multitargeted inhibitor against SARS-CoV-2: an in-silico study. J. Biomol. Struct. Dyn. 41, 4013–4023. 10.1080/07391102.2022.2060308 35451934

[B8] Ahmad S. Raza K. (2023). Identification of 5-nitroindazole as a multitargeted inhibitor for CDK and transferase kinase in lung cancer: a multisampling algorithm-based structural study. Mol. Divers. 28, 1189–1202. 10.1007/s11030-023-10648-0 37058176

[B9] Ahmad S. Sayeed S. Bano N. Sheikh K. Raza K. (2022b). In-silico analysis reveals Quinic acid as a multitargeted inhibitor against cervical cancer. J. Biomol. Struct. Dyn. 41, 9770–9786. 10.1080/07391102.2022.2146202 36379678

[B10] Ahmad S. Singh A. P. Bano N. Raza K. Singh J. Medigeshi G. R. (2024b). Integrative analysis discovers Imidurea as dual multitargeted inhibitor of CD69, CD40, SHP2, lysozyme, GATA3, cCBL, and S-cysteinase from SARS-CoV-2 and *M. tuberculosis* . Int. J. Biol. Macromol. 270, 132332. 10.1016/j.ijbiomac.2024.132332 38768914

[B11] Ahmad S. Singh V. Gautam H. K. Raza K. (2024c). Multisampling-based docking reveals Imidazolidinyl urea as a multitargeted inhibitor for lung cancer: an optimisation followed multi-simulation and *in-vitro* study. J. Biomol. Struct. Dyn. 42, 2494–2511. 10.1080/07391102.2023.2209673 37154501

[B12] Al Khzem A. H. Alturki M. S. Almuzaini O. K. Wali S. M. Almaghrabi M. Aldawsari M. F. (2025a). Isoetin from isoetaceae exhibits superior pentatransferase inhibition in breast cancer: comparative computational profiling with FDA-approved tucatinib. Pharmaceuticals 18, 662. 10.3390/ph18050662 40430480 PMC12115012

[B13] Al Khzem A. H. Gomaa M. S. Alturki M. S. Tawfeeq N. Sarafroz M. Alonaizi S. M. (2024). Drug repurposing for cancer treatment: a comprehensive review. Int. J. Mol. Sci. 25, 12441. 10.3390/ijms252212441 39596504 PMC11595001

[B14] Al Khzem A. H. Shoaib T. H. Mukhtar R. M. Alturki M. S. Gomaa M. S. Hussein D. (2025b). Repurposing FDA-approved agents to develop a prototype *Helicobacter pylori* shikimate kinase (HPSK) inhibitor: a computational approach using virtual screening, MM-GBSA calculations, md simulations, and DFT analysis. Pharmaceuticals 18, 174. 10.3390/ph18020174 40005988 PMC11858459

[B15] Aram E. Moeni M. Abedizadeh R. Sabour D. Sadeghi-Abandansari H. Gardy J. (2022). Smart and multi-functional magnetic nanoparticles for cancer treatment applications: clinical challenges and future prospects. Nanomaterials 12, 3567. 10.3390/nano12203567 36296756 PMC9611246

[B16] Ban T. Ohue M. Akiyama Y. (2018). Multiple grid arrangement improves ligand docking with unknown binding sites: application to the inverse docking problem. Comput. Biol. Chem. 73, 139–146. 10.1016/j.compbiolchem.2018.02.008 29482137

[B17] Baxter-Parker G. (2019). Induction of the innate immune response by physical trauma and infection: quantification through analysis of biochemical markers.

[B18] Bochevarov A. D. Harder E. Hughes T. F. Greenwood J. R. Braden D. A. Philipp D. M. (2013). Jaguar: a high‐performance quantum chemistry software program with strengths in life and materials sciences. Int. J. Quantum Chem. 113, 2110–2142. 10.1002/qua.24481

[B19] Bowers K. J. Chow E. Xu H. Dror R. O. Eastwood M. P. Gregersen B. A. (2006). “Scalable algorithms for molecular dynamics simulations on commodity clusters,” in Proceedings of the 2006 ACM/IEEE conference on supercomputing, 84–es.

[B20] Brøchner A. C. Toft P. (2009). Pathophysiology of the systemic inflammatory response after major accidental trauma. Scand. J. trauma, Resusc. Emerg. Med. 17, 43–10. 10.1186/1757-7241-17-43 19754938 PMC2757019

[B21] Bulters D. Gaastra B. Zolnourian A. Alexander S. Ren D. Blackburn S. L. (2018). Haemoglobin scavenging in intracranial bleeding: biology and clinical implications. Nat. Rev. Neurol. 14, 416–432. 10.1038/s41582-018-0020-0 29925923

[B22] Carbó R. Riera J. M. (2012). A general SCF theory. Springer Science and Business Media.

[B23] Chandrasekaran B. Abed S. N. Al-Attraqchi O. Kuche K. Tekade R. K. (2018). “Computer-aided prediction of pharmacokinetic (ADMET) properties,” in Dosage form design parameters (Elsevier), 731–755.

[B24] Ciccolini J. Mercier C. Dahan L. André N. (2011). Integrating pharmacogenetics into gemcitabine dosing—time for a change? Nat. Rev. Clin. Oncol. 8, 439–444. 10.1038/nrclinonc.2011.1 21304503

[B25] Costa A. Scholer-Dahirel A. Mechta-Grigoriou F. (2014). “The role of reactive oxygen species and metabolism on cancer cells and their microenvironment,” in Seminars in cancer biology (Elsevier), 23–32. 10.1016/j.semcancer.2013.12.007 24406211

[B26] Delanghe J. R. Delrue C. Speeckaert R. Speeckaert M. M. (2024). Unlocking the link between haptoglobin polymorphism and noninfectious human diseases: insights and implications. Crit. Rev. Clin. Laboratory Sci. 61, 275–297. 10.1080/10408363.2023.2285929 38013410

[B27] De Oliveira J. Denadai M. B. Costa D. L. (2022). Crosstalk between heme oxygenase-1 and iron metabolism in macrophages: implications for the modulation of inflammation and immunity. Antioxidants 11, 861. 10.3390/antiox11050861 35624725 PMC9137896

[B28] Dobryszycka W. (1997). Biological functions of haptoglobin-new pieces to an old puzzle. Eur. J. Clin. Chem. Clin. Biochem. 35, 647–654. 9352226

[B29] Dunphy K. O’mahoney K. Dowling P. O’gorman P. Bazou D. (2021). Clinical proteomics of biofluids in haematological malignancies. Int. J. Mol. Sci. 22, 8021. 10.3390/ijms22158021 34360786 PMC8348619

[B30] Famuyiwa S. O. Ahmad S. Fakola E. G. Olusola A. J. Adesida S. A. Obagunle F. O. (2023). Comprehensive computational studies of naturally occurring kuguacins as antidiabetic agents by targeting visfatin. Chem. Afr. 6, 1415–1427. 10.1007/s42250-023-00604-8

[B31] Gáll T. Balla G. Balla J. (2019). Heme, heme oxygenase, and endoplasmic reticulum stress—a new insight into the pathophysiology of vascular diseases. Int. J. Mol. Sci. 20, 3675. 10.3390/ijms20153675 31357546 PMC6695876

[B32] Galy B. Conrad M. Muckenthaler M. (2024). Mechanisms controlling cellular and systemic iron homeostasis. Nat. Rev. Mol. Cell. Biol. 25, 133–155. 10.1038/s41580-023-00648-1 37783783

[B33] Gbotosho O. T. Kapetanaki M. G. Kato G. J. (2021). The worst things in life are free: the role of free heme in sickle cell disease. Front. Immunol. 11, 561917. 10.3389/fimmu.2020.561917 33584641 PMC7873693

[B34] Hamilton T. P. Pulay P. (1986). Direct inversion in the iterative subspace (DIIS) optimization of open‐shell, excited‐state, and small multiconfiguration SCF wave functions. J. Chem. Phys. 84, 5728–5734. 10.1063/1.449880

[B35] He B. Huang Z. Huang C. Nice E. C. (2022). Clinical applications of plasma proteomics and peptidomics: towards precision medicine. PROTEOMICS–Clinical Appl. 16, 2100097. 10.1002/prca.202100097 35490333

[B36] Jacobson M. P. Pincus D. L. Rapp C. S. Day T. J. Honig B. Shaw D. E. (2004). A hierarchical approach to all‐atom protein loop prediction. Proteins Struct. Funct. Bioinforma. 55, 351–367. 10.1002/prot.10613 15048827

[B37] Jorgensen W. L. Tirado-Rives J. (1988). The OPLS [optimized potentials for liquid simulations] potential functions for proteins, energy minimizations for crystals of cyclic peptides and crambin. J. Am. Chem. Soc. 110, 1657–1666. 10.1021/ja00214a001 27557051

[B38] Kaczor A. A. Zięba A. Matosiuk D. (2024). The application of WaterMap-guided structure-based virtual screening in novel drug discovery. Expert Opin. Drug Discov. 19, 73–83. 10.1080/17460441.2023.2267015 37807912

[B39] Karwasra R. Ahmad S. Bano N. Qazi S. Raza K. Singh S. (2022). Macrophage-targeted punicalagin nanoengineering to alleviate methotrexate-induced neutropenia: a molecular docking, DFT, and MD simulation analysis. Molecules 27, 6034. 10.3390/molecules27186034 36144770 PMC9505199

[B40] Knutson M. D. (2017). Iron transport proteins: gateways of cellular and systemic iron homeostasis. J. Biol. Chem. 292, 12735–12743. 10.1074/jbc.r117.786632 28615441 PMC5546014

[B41] Lipinski C. A. (2004). Lead-and drug-like compounds: the rule-of-five revolution. Drug Discov. today Technol. 1, 337–341. 10.1016/j.ddtec.2004.11.007 24981612

[B42] Lu J. Wang Y. Yan M. Feng P. Yuan L. Cai Y. (2016). High serum haptoglobin level is associated with tumor progression and predicts poor prognosis in non-small cell lung cancer. Oncotarget 7, 41758–41766. 10.18632/oncotarget.9676 27248178 PMC5173094

[B43] Maestro S. (2022). Maestro. New York, NY: Schrödinger, LLC.

[B44] Mantovani A. Allavena P. Marchesi F. Garlanda C. (2022). Macrophages as tools and targets in cancer therapy. Nat. Rev. Drug Discov. 21, 799–820. 10.1038/s41573-022-00520-5 35974096 PMC9380983

[B45] Mark P. Nilsson L. (2001). Structure and dynamics of the TIP3P, SPC, and SPC/E water models at 298 K. J. Phys. Chem. A 105, 9954–9960. 10.1021/jp003020w

[B46] Mcdonald I. (1972). NpT-ensemble Monte Carlo calculations for binary liquid mixtures. Mol. Phys. 23, 41–58. 10.1080/00268977200100031

[B47] Montecinos L. Eskew J. D. Smith A. (2019). What is next in this “age” of heme-driven pathology and protection by hemopexin? An update and links with iron. Pharmaceuticals 12, 144. 10.3390/ph12040144 31554244 PMC6958331

[B48] Naryzny S. Legina O. (2021). Haptoglobin as a biomarker. Biochem. Mosc. Suppl. Ser. B Biomed. Chem. 15, 184–198. 10.1134/s1990750821030069 34422226 PMC8365284

[B49] Nielsen M. J. Moestrup S. K. (2009). Receptor targeting of hemoglobin mediated by the haptoglobins: roles beyond heme scavenging. Blood, J. Am. Soc. Hematol. 114, 764–771. 10.1182/blood-2009-01-198309 19380867

[B50] Olsson M. H. Søndergaard C. R. Rostkowski M. Jensen J. H. (2011). PROPKA3: consistent treatment of internal and surface residues in empirical p K a predictions. J. Chem. theory Comput. 7, 525–537. 10.1021/ct100578z 26596171

[B51] Orrico F. Laurance S. Lopez A. C. Lefevre S. D. Thomson L. Möller M. N. (2023). Oxidative stress in healthy and pathological red blood cells. Biomolecules 13, 1262. 10.3390/biom13081262 37627327 PMC10452114

[B52] Perepichka D. F. Bryce M. R. (2005). Molecules with exceptionally small HOMO-LUMO gaps. Angew. Chem. Int. Ed. 44, 5370–5373. 10.1002/anie.200500413 16034992

[B53] Qikprop S. (2022). Schrödinger release 2022. New York, USA: Maestro LLC.

[B54] Release S. (2022a). Desmond molecular dynamics system, DE Shaw research, New York, NY, 2022. New York, NY: Maestro-Desmond Interoperability Tools, Schrödinger.

[B55] Release S. (2022b). Glide: schrödinger. NY, USA: LLC.

[B56] Release S. (2022c). Jaguar. New York, NY: Schrödinger, LLC.

[B57] Release S. (2022d). New York, NY: LigPrep, Schrödinger, LLC.

[B58] Release S. (2022e). Receptor grid generation. New York, NY: Schrödinger, LLC.

[B59] Release S. (2022f). Schrödinger suite 2022 protein preparation wizard. New York, NY: Epik, Schrödinger, LLC.

[B60] Roh T. Ju S. Park S. Y. Ahn Y. Chung J. Nakano M. (2025). Fucosylated haptoglobin promotes inflammation via Mincle in sepsis: an observational study. Nat. Commun. 16, 1342. 10.1038/s41467-025-56524-3 39904983 PMC11794430

[B61] Schaer D. J. Schaer C. A. Buehler P. W. Boykins R. A. Schoedon G. Alayash A. I. (2006). CD163 is the macrophage scavenger receptor for native and chemically modified hemoglobins in the absence of haptoglobin. Blood 107, 373–380. 10.1182/blood-2005-03-1014 16189277

[B62] Schaer D. J. Vinchi F. Ingoglia G. Tolosano E. Buehler P. W. (2014). Haptoglobin, hemopexin, and related defense pathways—basic science, clinical perspectives, and drug development. Front. physiology 5, 415. 10.3389/fphys.2014.00415 PMC4211382 25389409

[B63] Schlegel H. B. (2011). Geometry optimization. Wiley Interdiscip. Rev. Comput. Mol. Sci. 1, 790–809. 10.1002/wcms.34

[B64] Shelley J. C. Cholleti A. Frye L. L. Greenwood J. R. Timlin M. R. Uchimaya M. (2007). Epik: a software program for pK a prediction and protonation state generation for drug-like molecules. J. computer-aided Mol. Des. 21, 681–691. 10.1007/s10822-007-9133-z 17899391

[B65] Singh R. Bhardwaj V. Upadhyay A. K. Kaur M. Garg S. Nagarwal A. (2025). Haptoglobin polymorphisms: translational insights and regenerative potential in disease management. Regen. Eng. Transl. Med., 1–14.40401122

[B66] Skytthe M. K. Graversen J. H. Moestrup S. K. (2020). Targeting of CD163+ macrophages in inflammatory and malignant diseases. Int. J. Mol. Sci. 21, 5497. 10.3390/ijms21155497 32752088 PMC7432735

[B67] Soares C. M. Teixeira V. H. Baptista A. M. (2003). Protein structure and dynamics in nonaqueous solvents: insights from molecular dynamics simulation studies. Biophysical J. 84, 1628–1641. 10.1016/s0006-3495(03)74972-8 PMC1302733 12609866

[B68] Telen M. J. Malik P. Vercellotti G. M. (2019). Therapeutic strategies for sickle cell disease: towards a multi-agent approach. Nat. Rev. Drug Discov. 18, 139–158. 10.1038/s41573-018-0003-2 30514970 PMC6645400

[B69] Tishkowski K. Gupta V. (2020). Erythrocyte sedimentation rate, 121–124. 10.1016/b978-0-323-43044-9.00011-x 32491417

[B70] Tripathi M. K. Ahmad S. Tyagi R. Dahiya V. Yadav M. K. (2022). “Fundamentals of molecular modeling in drug design,” in Computer Aided Drug Design (CADD): from ligand-based methods to structure-based approaches (Elsevier), 125–155.

[B71] Tuccinardi T. (2021). What is the current value of MM/PBSA and MM/GBSA methods in drug discovery? Expert Opin. drug Discov. 16, 1233–1237. 10.1080/17460441.2021.1942836 34165011

[B72] Van Avondt K. Nur E. Zeerleder S. (2019). Mechanisms of haemolysis-induced kidney injury. Nat. Rev. Nephrol. 15, 671–692. 10.1038/s41581-019-0181-0 31455889

[B73] Wang E. Sun H. Wang J. Wang Z. Liu H. Zhang J. Z. (2019). End-point binding free energy calculation with MM/PBSA and MM/GBSA: strategies and applications in drug design. Chem. Rev. 119, 9478–9508. 10.1021/acs.chemrev.9b00055 31244000

[B74] Wang T. Ashrafi A. Modareszadeh P. Deese A. R. Chacon Castro M. D. C. Alemi P. S. (2021). An analysis of the multifaceted roles of heme in the pathogenesis of cancer and related diseases. Cancers 13, 4142. 10.3390/cancers13164142 34439295 PMC8393563

[B75] Wang Y. Bellomo R. (2017). Cardiac surgery-associated acute kidney injury: risk factors, pathophysiology and treatment. Nat. Rev. Nephrol. 13, 697–711. 10.1038/nrneph.2017.119 28869251

[B76] Witte J. Mardirossian N. Neaton J. B. Head-Gordon M. (2017). Assessing DFT-D3 damping functions across widely used density functionals: can we do better? J. Chem. theory Comput. 13, 2043–2052. 10.1021/acs.jctc.7b00176 28394597

[B77] Yadav M. K. Ahmad S. Raza K. Kumar S. Eswaran M. Pasha Km M. (2022). Predictive modeling and therapeutic repurposing of natural compounds against the receptor-binding domain of SARS-CoV-2. J. Biomol. Struct. Dyn. 41, 1527–1539. 10.1080/07391102.2021.2021993 34974820

