# Correction: Integrative computational approaches identify haptoglobin inhibitors to modulate erythrocyte sedimentation rate in trauma-linked inflammatory and haematological malignancies

**DOI:** 10.3389/fchem.2025.1662748

**Published:** 2025-07-29

**Authors:** Abdulaziz H. Al Khzem, Shaban Ahmad

**Affiliations:** ^1^ Department of Pharmaceutical Chemistry, College of Pharmacy, Imam Abdulrahman Bin Faisal University, Dammam, Saudi Arabia; ^2^ Department of Computer Science, Jamia Millia Islamia, New Delhi, India

**Keywords:** haptoglobin, drug design and discovery, computational medicinal chemistry, molecular docking, binding free energy

Author Shaban Ahmad was erroneously assigned to the affiliation “Department of Veterinary and Animal Sciences, Faculty of Health and Medical Sciences, University of Copenhagen, Frederiksberg, Denmark”. This is in fact the author’s present affiliation and did not apply at the time of the study. The author’s primary email address (shaban184343@st.jmi.ac.in) was also missing from the Correspondence section.

The original article has been updated.

